# Decoding bull fertility *in vitro*: a proteomics exploration from sperm to blastocyst

**DOI:** 10.1530/REP-24-0296

**Published:** 2025-03-19

**Authors:** Andrea Fernández-Montoro, Emin Araftpoor, Tine De Coster, Daniel Angel-Velez, Marcel Bühler, Mohamed Hedia, Kris Gevaert, Ann Van Soom, Krishna Chaitanya Pavani, Katrien Smits

**Affiliations:** ^1^ Reproductive Biology Unit, Department of Internal Medicine, Reproduction and Population Medicine, Ghent University, Merelbeke, Belgium; ^2^ VIB-UGent Center for Medical Biotechnology, VIB, Ghent, Belgium; ^3^ Department of Biomolecular Medicine, Ghent University, Ghent, Belgium; ^4^ Research Group in Animal Sciences – INCA-CES, Universidad CES, Medellin, Colombia; ^5^ Theriogenology Department, Cairo University, Giza, Egypt; ^6^ Department for Reproductive Medicine, Ghent University Hospital, Ghent, Belgium

**Keywords:** embryo development, *in vitro* fertilization, male fertility, polyspermy, proteomics

## Abstract

**In brief:**

Bulls are selected for field fertility and semen quality, but traits such as polyspermy are not considered and can increase aneuploidy during *in vitro* embryo production. This study links bull-specific proteomic signatures to polyspermy and embryo quality, further refining bull selection criteria.

**Abstract:**

Male fertility plays a pivotal role in the success rates of *in vitro* embryo production. While livestock breeding programs rigorously select bulls according to their predicted field fertility, specific traits such as polyspermy rates are not routinely evaluated. Despite the known negative impact of polyspermy on embryo survival, the paternal factors involved remain unclear. In this study, we aimed to address this gap by evaluating the *in vitro* outcomes of four bulls, focusing on sperm motility, fertilization rates, polyspermy incidence, embryo development and quality. In addition, we analyzed the proteome profiles of sperm, 2–4 cell stage embryos and blastocysts derived from those bulls to identify potential molecular factors associated with male fertility. Bulls with comparable sperm motility parameters displayed varying *in vitro* fertilization outcomes. Notably, the bull with the highest polyspermy rate achieved blastocyst rates similar to those of bulls with lower polyspermy rates. The number of apoptotic cells in the blastocysts was bull-dependent. Proteomic analysis revealed bull-specific signatures in sperm and blastocysts, with no differences at the 2–4 cell stage. Differences in the sperm proteome suggested that bull-dependent penetration and polyspermy rates might be associated with the ability of the sperm to undergo capacitation and acrosomal reaction. At the blastocyst level, the bull with the highest polyspermy rates produced lower quality blastocysts due to imbalances in key proteins and pathways for embryo development. In conclusion, bulls with similar blastocyst rates may differ in polyspermy rates and resulting embryo quality underscoring the importance of careful bull selection for *in vitro* embryo production.

## Introduction

The number of bovine embryos produced *in vitro* has triplicated in the past decade (457,455 transferable embryos in 2012 vs 1,616,971 in 2022) (Perry 2013, Viana 2023). Yet, the efficacy of *in vitro* embryo production (IVP) in livestock animals rarely surpasses the 30–40% threshold, indicating that a substantial number of oocytes do not successfully develop after *in vitro* fertilization (IVF) and culture (Ferré *et al.* 2019). Variation in male fertility is one of the factors contributing to the high variability of IVP outcomes (Sattar *et al.* 2011). To categorize bulls’ fertility for IVP programs, breeding soundness evaluation, field trials and *in vitro* assessment of sperm characteristics or blastocyst rates are performed (Gillan *et al.* 2008, Kaya & Memili 2016). However, specific traits such as polyspermy, defined as the penetration of more than one spermatozoon into the oocyte, are not routinely analyzed. While easily detected in translucent murine and human zygotes, the lipid-rich, opaque cytoplasm of bovine oocytes prevents the visualization of pronuclei and metaphase plates. Although the occurrence of abnormal fertilization can be assessed in bovine zygotes by specific staining (Fernández-Montoro *et al.* 2024), the extra testing of bulls before using them for *in vitro* production of embryos is often neglected. However, it is important to evaluate each bull for polyspermic fertilization, since polyspermy instigates whole-genome errors, resulting in mosaic embryos and leading to low calving rates due to embryonic arrest, implantation failure or fetal mortality (Snook *et al.* 2011, De Coster *et al.* 2022).

In pigs, IVP is burdened by polyspermy (up to 65%) (Wang *et al.* 1998, Xia *et al.* 2001); thus, they are an ideal model for studying the developmental consequences of polyspermy (Funahashi 2003, Grupen 2014). Penetration and polyspermy rates vary among breeds, individual boars, ejaculates from the same boar and fractions within the same ejaculate (Matás *et al.* 1996, Xu *et al.* 1996*a*,*b*, Suzuki *et al.* 2003). Polyspermic porcine embryos can reach similar blastocyst rates compared to normal diploid embryos but with reduced inner cell mass nuclei (Han *et al.* 1999, Koo *et al.* 2005). In cattle, differences in penetration rates have been described among bulls, but polyspermy rates were not different (Ohgoda *et al.* 1988, Saeki *et al.* 1995, Alomar *et al.* 2006). Other studies found variations in penetration and polyspermy rates among bulls (Marquant-Le Guienne *et al.* 1990), whereas conflicting data were found about their influence on embryo development (Long *et al.* 1994, Kreysing *et al.* 1997, Alomar *et al.* 2008). Therefore, more research is needed to clarify a potential bull effect on normal vs polyspermic fertilization and its correlation with subsequent embryo development.

Sperm provides a complex population of RNA molecules and proteins to the oocyte that might regulate early embryo development (Jodar 2019, Vallet-Buisan *et al.* 2023). An integrative analysis of transcriptomic and proteomic data from human sperm, oocytes and early embryos identified eleven blastocyst proteins that most likely originated from the translation of intact sperm mRNAs during early embryogenesis (Castillo *et al.* 2018). Furthermore, other studies have shown associations between bull fertility and the transcriptome and proteome profiles of sperm (Peddinti *et al.* 2008, Gross *et al.* 2019), and with the transcriptome profile of four-cell (Lockhart *et al.* 2023) and preimplantation embryos (Kropp *et al.* 2017). The aforementioned studies based the fertility status of the sires on their capacity to produce blastocysts *in vitro* or their conception rates. However, there is no information on the paternal contributions toward polyspermy-related developmental defects and the underlying proteomic changes.

Therefore, this study aimed i) to compare four bulls to determine individual variability in sperm motility, fertilization, polyspermy embryo development and quality and correlations between these variables and ii) to provide insights into the molecular traits of sperm from bulls with different *in vitro* outcomes and their resulting early and preimplantation embryos by proteomics. We hypothesized that polyspermy rates may be bull specific, possibly affecting embryo development and that these differences would be reflected in the protein profiles.

## Materials and methods

**Figure 1:**
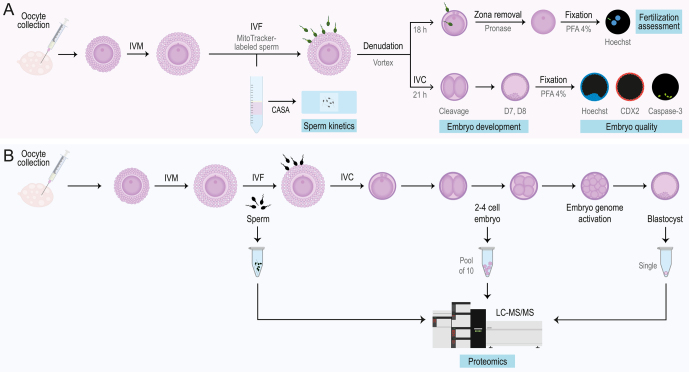
Experimental design. (A) In Experiment 1, four bulls were subjected to *in vitro* embryo production (IVP) to determine the fertilization status of the zygotes, embryo development (tracked on day 2, 7 and 8 post-insemination) and embryo quality. Additionally, sperm motility parameters were examined. (B) In experiment 2, differentially expressed proteins were analyzed in sperm, 2-4 cell embryos and blastocysts from one bull with high polyspermy rates and three other bulls with lower polyspermy rates. IVM, *in vitro* maturation; IVF, *in vitro* fertilization; IVC, *in vitro* culture; PNA, peanut agglutinin; PFA, paraformaldehyde; D7, day 7; D8, day 8.

Tissue Culture Media (TCM)-199 with Earle’s salts, TCM-199 with Hanks’ salts and Dulbecco’s phosphate-buffered saline (DPBS) were purchased from Gibco™ Thermo Fisher Scientific (USA). If not specified, all other products were obtained from Sigma-Aldrich (Belgium). All media were filtered before use (0.22 μm; Novolab, Belgium). Two experiments were conducted (Fig. 1), where experiment 1 determined the fertilization status of the zygotes, embryo development and embryo quality, and experiment 2 investigated the proteomic profile in sperm, 2–4 cell embryos and blastocysts.

### Oocyte collection and IVF

Slaughterhouse-derived ovaries were collected and transported within 2 h to the laboratory in an insulated box (Wydooghe *et al.* 2014). The ovaries were disinfected (96% ethanol) and washed (three times) in warm saline containing 25 μg/mL kanamycin. Follicles (3–8 mm in diameter) were punctured with an 18 G needle attached to a 10 mL syringe. Compact cumulus–oocyte complexes (COCs; at least three layers) with homogenous ooplasm were collected, washed in warm HEPES-Tyrode’s Albumin Lactate Pyruvate media (HEPES-TALP) and *in vitro* matured (IVM; TCM-199 Earle’s salts supplemented with 20 ng/mL epidermal growth factor and 50 ppm gentamicin) in groups of 60 in 500 μL IVM medium for 22 h at 38.5°C in 5% CO_2_ in humidified air.

After IVM, the oocytes were washed and co-incubated with the spermatozoa (IVF) at 38.5°C in 5% CO_2_ in humidified air. Four Holstein–Friesian bulls (*Bos taurus*) of proven fertility, aged 4–5 years, were used. All bulls presented a non-return rate of 56 days higher than 50% (bull 1 = 58.47, bull 2 = 60.82, bull 3 = 58.29 and bull 4 = 64.87). Three frozen-thawed straws per bull were washed in a discontinuous gradient of 45/90% Percoll® (GE Healthcare Biosciences, Sweden). The sperm concentration was adjusted to a final concentration of 1 × 10^6^ spermatozoa/mL using IVF–TALP medium enriched with BSA (Sigma-Aldrich A8806; 6 mg/mL) and heparin (20 μg/mL).

### Motility assessment by computer-assisted sperm analysis (CASA)

In each replicate and for each independent bull, a pool of three frozen-thawed straws from the same ejaculate was used and the sperm motility parameters were analyzed in triplicates with the CASA system (Hamilton-Thorne motility analyzer Ceros version 12.3 days; Hamilton-Thorne Research, USA).

### Evaluation of the fertilization status

For experiment 1, the sperm pellets were labeled by washing in 5 mL IVF–TALP medium enriched with BSA (6 mg/mL) and 200 nmol/L mitochondria-specific stain (MitoTracker® Green FM, Molecular Probes, Belgium) dissolved in DMSO (Sigma-Aldrich, Belgium) (Fernández-Montoro *et al.* 2024). After 18 h of IVF, half of the presumed zygotes (bull 1: *n* = 261, bull 2: *n* = 225, bull 3: *n* = 272 and bull 4: *n* = 121, 13 replicates) were vortexed for 4 min in 2.5 mL HEPES-TALP and incubated with 0.03% pronase (protease from *S. griseus*, Sigma-Aldrich, Belgium) in TCM-199 and then washed in 10% fetal bovine serum in TCM-199. A stripper pipette equipped with 135 and 170 μm capillaries (ORIGIO, Cooper Surgical, USA) was used for zona removal in Ca^2+^/Mg^2+^-free DPBS supplemented with 0.1% polyvinylpyrrolidone (PVP), and the zona-free presumed zygotes were fixed in 4% paraformaldehyde in DPBS for at least 1 h. Subsequently, the presumed zygotes were washed twice in DPBS with 0.1% PVP to remove the fixative, incubated for 15 min with 3.2 μmol/L Hoechst 33342 solution in DPBS with 0.1% PVP and washed four times in DPBS with 0.1% PVP. The presumed zygotes were then mounted on siliconized glass slides using 1,4-diazabicyclo[2,2,2]octane (DABCO). The presence of a MitoTracker Green FM-positive sperm tail and Hoechst-labeled DNA from the pronucleus was used to determine the fertilization status of the presumed zygotes, since it was not possible to evaluate that in living zygotes due to the opaque cytoplasm. Penetrated zygotes are those with at least one sperm tail in the ooplasm. Zygotes exhibiting two pronuclei and one tail were considered monospermic, while zygotes with more than two tails were classified as polyspermic. The condensation status of the DNA was determined. Small and intensively stained nuclei were categorized ‘condensed’, while round and less intense nuclei were labeled ‘decondensed’. Pronuclei were considered apposed when they were touching or extremely close to each other, and non-apposed when they were separated (Fernández-Montoro *et al.* 2024). The percentage of monospermic zygotes from the total presumed zygotes (monospermic/polyspermic/not fertilized) was defined as IVF efficiency.

### *In vitro* embryo culture

After 21 h of IVF, the remaining presumed zygotes (bull 1: *n* = 318, bull 2: *n* = 296, bull 3: *n* = 272 and bull 4: *n* = 163, 13 replicates) were vortexed for 3 min in 2.5 mL HEPES-TALP and cultured under oil (SAGE oil for tissue culture, ART-4008-5P, Cooper Surgical Company) in groups of 25–30 in 50 μL droplets of synthetic oviductal fluid, enriched with 4 mg/mL BSA, non-essential and essential amino acids (SOFaa) and ITS (5 μg/mL insulin, 5 μg/mL transferrin and 5 ng/mL selenium), at 38.5°C for 8 days in 5% CO_2_, 5% O_2_ and 90% N_2_. Cleavage rate was recorded 45 h post-fertilization and blastocyst development rates were evaluated on day 7 and day 8 post-fertilization.

### Embryo quality assessment

Using differential apoptotic staining (Wydooghe *et al.* 2011), embryo quality was assessed in four IVP replicates of experiment 1. Expanded day 8 blastocysts were fixed in 2% paraformaldehyde for 20 min and stored in 0.5% (w/v) BSA in DPBS at 4°C until further processing. Blastocysts were incubated with mouse anti-CDX2 primary antibody (Biogenex, USA), followed by goat anti-mouse Texas Red secondary antibody (20 μg/mL in blocking solution, Molecular Probes, Belgium), to rabbit active caspase-3 primary antibody (0.768 ng/mL, Cell Signaling Technology, the Netherlands), followed by goat anti-rabbit FITC secondary antibody (10 μg/mL in blocking solution, Molecular Probes), and to Hoechst 33342 (50 μg/mL in DPBS/BSA). A single observer examined samples under fluorescence microscopy (Leica DM 5500 B, Germany; 40×). Total cell number (TCN), the number of trophectoderm cells (TE) and apoptotic cells (AC) and the calculation of the inner cell mass cells (ICM = TCN – TE), the ICM/TCN ratio and the AC ratio (AC/TCN) were recorded.

### Statistical analysis of sperm parameters, fertilization assessment, embryo development and quality

Results are expressed as the least-square mean, probability or means ± standard error. Statistical analyses were performed in the RStudio version 2024.04.1+748 (R Core Team, Austria). A Shapiro–Wilk’s test was used and abnormally distributed data were transformed whenever possible. Differences between groups were assessed using the Tukey’s post hoc test (general and linear mixed models) in normally distributed data. Multiple comparisons were performed by the Kruskal–Wallis test, followed by Benjamini and Hochberg for abnormally distributed ones. Bull’s effect on sperm motility, fertilization status and embryo development was assessed by generalized mixed effect models that were fit with the replicate as a random effect. Bull’s effect on the number of sperm tails counted in polyspermic zygotes and blastocyst quality parameters was analyzed by linear mixed effect models with the replicate or sample size of the blastocyst as a random effect, respectively. Pearson correlation coefficients were calculated between motility parameters, fertilization status and developmental parameters. Significance and tendency levels were set at *P* < 0.05 and *P* < 0.1, respectively.

### Proteomics sample preparation and data analysis

Detailed information on proteomics sample preparation and data analysis can be found in the supplementary methods file. Sperm cell pellets from IVP were lysed in an SDS-containing lysis buffer, followed by sonication. Supernatants were reduced and alkylated and further processed using S-Trap™ mini columns (ProtiFi C02-mini, USA), following the manufacturer’s instructions. Peptides were oxidized with 3% H_2_O_2_ and cleaned up using ZipTips (Agilent, Belgium).

To identify a possible bull effect on the proteome of embryos before and after embryonic genome activation, pools of ten 2–4 cell stage embryos or single blastocysts were collected in 2 μL DPBS in a twin.tec® PCR Plate 384 LoBind® (Eppendorf, Germany) and stored at −80°C. Samples were lysed by three freeze-thaw cycles, followed by in-solution digestion with a Trypsin/Lys-C-based digestion mix (Promega, USA). Digested peptides were acidified with 5% TFA and loaded onto Evotips (Evosep, Denmark), according to manufacturer’s instructions.

For sperm samples, 500 ng peptide material was injected via a Vanquish™ Neo UHPLC System into an Orbitrap Exploris 240 mass spectrometer (Thermo Scientific, Germany) operated in data-independent acquisition (DIA)-mode. Peptides derived from 2 to 4-cell embryo pools or single blastocyst digests were separated on an Evosep system and analyzed on-line by a timsTOF SCP mass spectrometer (Bruker Daltonics, Germany) operated in data-independent parallel accumulation serial fragmentation (DIA-PASEF) mode.

Sperm raw data files were searched using the DIA-NN v1.8.1 (Demichev *et al.* 2020, 2022). The search engine was supplied with the bovine reference proteome (*Bos taurus* UP000009136 with 23,836 entries; one protein sequence per gene) for library generation, allowing for one missed cleavage using Trypsin/P as the protease, up to two variable modifications, with N-terminal methionine excision, methionine oxidation and N-terminal acetylation set as variable modifications and cysteine carbamidomethylation set as a fixed modification. Peptide length and charge ranges were set to 7–30 and 1–4, respectively. Precursor and fragment ion ranges were set to respectively 400–900 and 200–1,800, respectively. Precursors were filtered at 1% false discovery rate, with robust LC (high accuracy), cross-run retention time-based normalization and match between runs enabled. Embryo raw data files were searched using identical settings as described above, with the following exceptions: cysteine carbamidomethylation was disabled, the precursor mass range was set to 400–1,000 and robust LC (high precision) was enabled.

Downstream data analysis was performed with the R version 4.3.3 within the RStudio environment version 2023.12.0+369. Statistical analysis was performed using the MSqRob2 and QFeatures packages (Goeminne *et al.* 2020, Gatto & Vanderaa 2024). Plotting was performed using the ggplot2 package (Wickham 2016). Gene set enrichment analysis (GSEA) analyses were performed using the WebGestaltR package (Liao *et al.* 2019).

## Results

### Assessment of sperm motility

Sperm cell motilities were not significantly (*P* > 0.05) different between bulls (Table 1). However, bull 4 exhibited higher straightness (STR = VSL/VAP) than bull 2 (*P* = 0.032), higher wobble movement (WOB = VAP/VCL) than bulls 1 (*P* = 0.008) and 2 (*P* = 0.016) and lower amplitude of lateral head (ALH) displacement than bulls 1 (*P* = 0.009) and 2 (*P* = 0.007).

**Table 1:** Assessment of sperm motility from individual bulls. Results are expressed as the least square means ± standard error (LSM ± SE).

Motility parameters	Bull 1	Bull 2	Bull 3	Bull 4
Total motility, %	58.30 ± 5.34	59.20 ± 5.34	47.0 ± 5.98	56.60 ± 8.45
Progressive motility, %	40.60 ± 4.10	37.90 ± 4.10	30.10 ± 4.58	41.30 ± 6.48
VCL, μm/s	108.40 ± 4.73	111.40 ± 4.73	97.40 ± 5.29	109.40 ± 7.48
VSL, μm/s	51.70 ± 4.32	52.80 ± 4.32	50.20 ± 4.83	69.70 ± 6.83
VAP, μm/s	64.10 ± 4.40	67.20 ± 4.40	62.10 ± 4.92	80.20 ± 6.95
LIN, %	47.40 ± 2.48	46.80 ± 5.34	50.8 ± 2.78	63.4 ± 3.93
STR, %	80.20 ± 1.61^ab^	77.80 ± 1.61^a^	80.0 ± 1.61^ab^	86.6 ± 2.55^b^
WOB, %	58.90 ± 2.11^a^	60.0 ± 2.11^a^	63.40 ± 2.35^ab^	72.7 ± 3.33^b^
ALH, μm	4.72 ± 0.19^a^	4.75 ± 0.214^a^	4.05 ± 0.21^ab^	3.48 ± 0.30^b^
BCF	9.11 ± 0.47	9.48 ± 0.47	9.53 ± 0.53	11.22 ± 0.75

Different superscripts within rows (a and b) represent statistical differences (*P* < 0.05) between bulls.

VCL, curvilinear velocity; VSL, straight-line velocity; VAP, average path velocity; LIN, linearity; STR, straightness; WOB, wobble; ALH, amplitude of lateral head displacement; BCF, beat-cross frequency.

### Polyspermy rates are bull-specific

**Figure 2:**
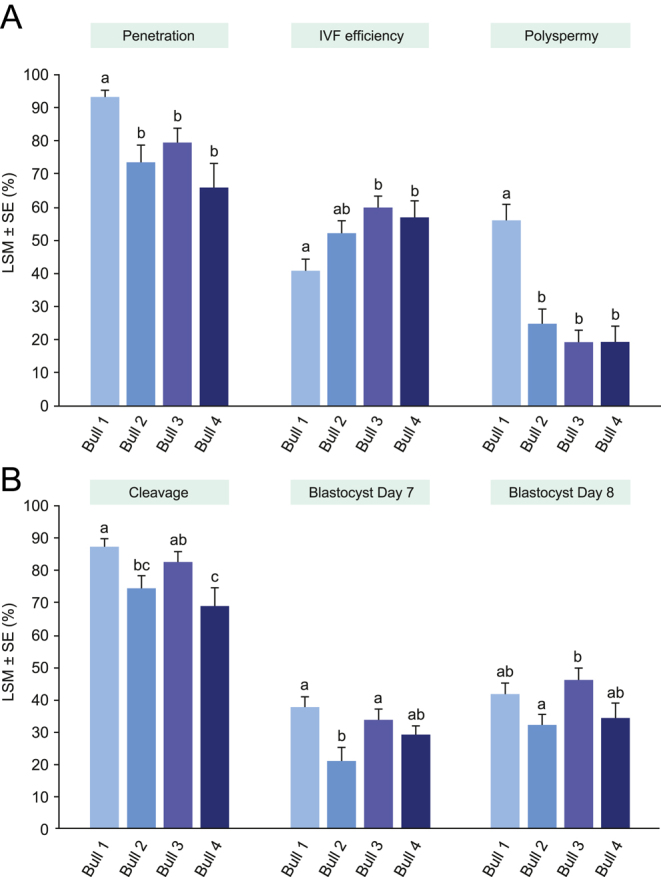
(A) Fertilization status from zygotes generated with sperm of four different bulls. Penetration rate is expressed as the number of zygotes penetrated by at least one sperm cell over the presumed zygotes. IVF efficiency is calculated as the number of monospermic zygotes out of the number of presumed zygotes. Polyspermy rate is calculated as the number of zygotes penetrated by more than one sperm cell over the number of penetrated zygotes. (B) Embryo development after fertilization of oocytes with four different bulls. Cleavage, day 7 and day 8 blastocyst rates are determined as a percentage over the presumed zygotes. Different superscripts (a, b, c; *, **; +, ++) indicate statistical differences (P < 0.05) between groups. Results are expressed as least square means ± standard error (LSM ± SE).

There was a significant bull effect on the rates of penetration, IVF efficiency, polyspermy and decondensation (*P* < 0.001; Fig. 2A, Table S2 (see section on Supplementary materials given at the end of the article)). Specifically, bull 1 presented a higher penetration rate than bull 2 (*P* < 0.001), bull 3 (*P* < 0.001) and bull 4 (*P* < 0.001); lower IVF efficiency than bull 3 (*P* < 0.001) and bull 4 (*P* = 0.028); and higher polyspermy rate compared to bull 2 (*P* < 0.001), bull 3 (*P* < 0.001) and bull 4 (*P* < 0.001). According to these results, bulls were classified as high polyspermy (bull 1) and low polyspermy (bulls 2, 3 and 4). Zygotes from bull 3 showed a higher decondensation rate compared to bull 1 (*P* < 0.001) and bull 4 (*P* = 0.007), while bull 2 had a higher decondensation rate than bull 1 (*P* = 0.012). Pronuclei apposition rate did not differ among bulls (*P* = 0.658) (Table S2).

### High and low polyspermy bulls can yield similar blastocyst rates

There was a significant bull effect on the cleavage rate (*P* < 0.001) and percentages of day 7 (*P* < 0.001) and day 8 (*P* = 0.004) blastocyst (Fig. 2B). While bull 4 produced fewer cleaved embryos than bull 1 (*P* < 0.001) and bull 3 (*P* = 0.042), bull 1 presented higher cleavage rates than bull 2 (*P* < 0.001). Bull 2 had a lower day 7 blastocyst rate compared to bull 1 (*P* < 0.001) and bull 3 (*P* = 0.005). Bull 2 had a lower day 8 blastocyst rate compared to bull 3 (*P* = 0.005). No significant differences (*P* > 0.05) were found between bulls for early, normal, expanded, hatching or hatched day 8 embryos.

### Embryo quality is not necessarily linked to embryo development

There was a significant effect of the bull on AC and the AC/TCN ratio (*P* < 0.001) (Table 2). Pairwise comparisons showed that embryos resulting from fertilization with bull 4 presented lower AC and AC/TCN ratio compared to bull 1 (*P* = 0.01 and *P* < 0.001, respectively), bull 2 (*P* = 0.006 and 0.01, respectively) and bull 3 (both *P* < 0.001). In contrast, there was no significant effect of the bull on TCN, TE, ICM and ICM/TCN (*P* > 0.05), but bull 1 tended to have lower ICM cell number than bull 2 (*P* = 0.09) and lower ICM/TCN than bull 3 (*P* = 0.06).

**Table 2:** Bull effect on day 8 embryo quality determined by differential staining. Results are expressed as the least-square mean ± SE, except for AC/TCN, for which results are expressed as the means ± SE.

Bull	Blastocysts, *n*	Cells, *n*				ICM/TCN ratio, %	AC/TCN ratio, %
		TCN	TE	ICM	AC		
1	44	211 ± 28.9	135 ± 24.3	72.1 ± 0.1	20.4 ± 1.2^a^	36.7 ± 3.3	11.2 ± 5.4^a^
2	25	241 ± 30.1	141 ± 24.9	95.1 ± 0.2	22.4 ± 1.2^a^	42.1 ± 3.7	11.1 ± 6.4^a^
3	46	223 ± 28.9	130 ± 24.3	90.3 ± 0.1	22.9 ± 1.2^a^	43.0 ± 3.4	11.9 ± 6.6^a^
4	28	228 ± 29.7	138 ± 24.7	86.3 ± 0.2	12.9 ± 1.2^b^	40.4 ± 3.6	6.74 ± 3.9^b^

TCN, total cell number; TE, trophectoderm cells; ICM, inner cell mass; AC apoptotic cells.

Different superscripts per column (a and b) represent statistical differences (*P* < 0.05) among groups.

### Correlation analysis

To evaluate possible associations between sperm motility, fertilization and embryo development, we performed a correlation analysis. The penetration rate was positively correlated with polyspermy (*P* < 0.001) and blastocyst rate at day 7 (*P* = 0.001). Polyspermy rate was positively correlated with cleavage and (*P* = 0.004) blastocyst formation at day 7 (*P* = 0.006). Day 7 blastocyst rate was positively correlated with progressive motility (*P* = 0.008); correlation coefficients are depicted in Table S3.

### Sperm and blastocyst proteomes vary among bulls

**Figure 3:**
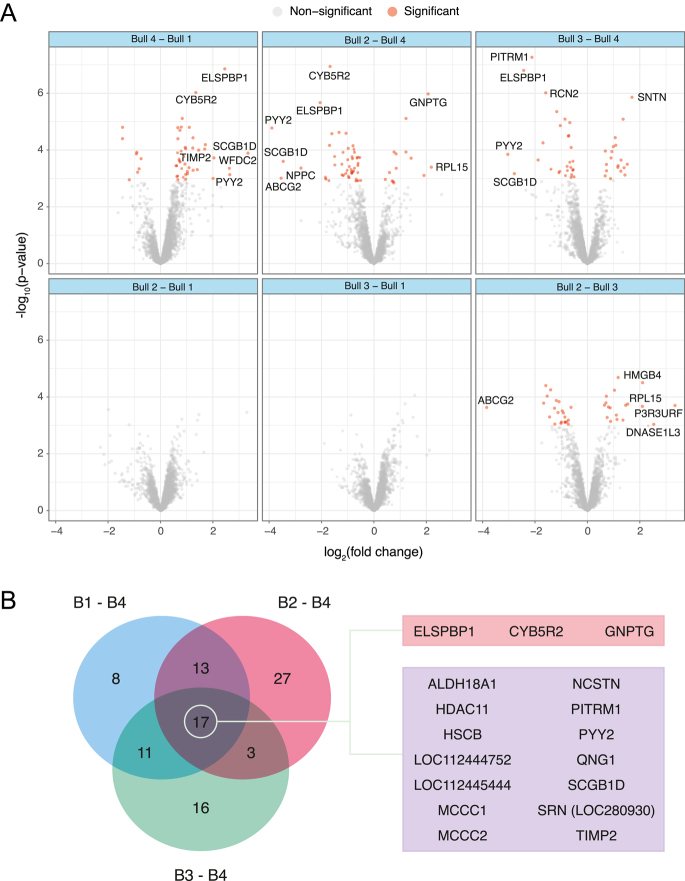
Comparative proteomics analysis of sperm from different bulls. (A) Volcano plots showing differentially abundant (DA) proteins (adjusted *P*-value < 0.05) in pairwise comparisons between bulls. Each point represents a protein, with the X-axis denoting log_2_ fold change and the Y-axis denoting -log_10_ *P*-value. (B) Venn diagram illustrating DA proteins in comparisons against bull 4, with the center showing proteins common across all comparisons. Genes coding for upregulated proteins are highlighted in red, and those coding for downregulated proteins are in purple. Only proteins with an absolute log_2_ fold change of 1.2 or greater were plotted.

**Figure 4:**
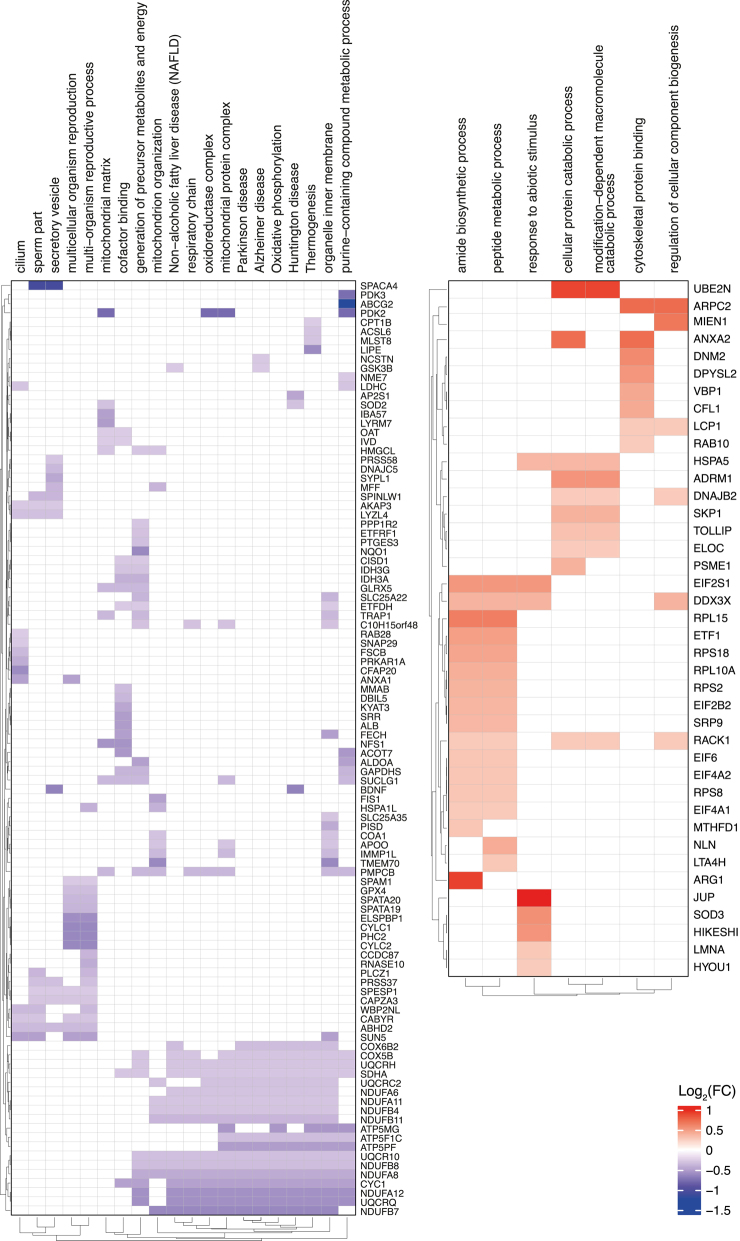
Gene Set Enrichment Analysis (GSEA) of sperm from bull 1 compared to bulls 2, 3 and 4. The figure presents GSEA results using heat maps, including the list of genes (right side of each heat map) involved in the different pathways (on top of the heat map). The left side shows heat maps of down-regulated pathways in bull 1 (purple) while the right side shows heat maps of up-regulated pathways (red). Only proteins with an absolute log_2_ fold change of 1.3 or greater were plotted.

In total, 2,120 proteins were identified in bull’s sperm. Bull 4 (higher blastocyst quality) had the highest amount of differentially abundant (DA) proteins against the three others (Figs 3A and S2, Table S4), with 72 DA proteins identified. Specifically, 49 proteins were DA between bull 4 and bull 1 (41 more and eight less abundant, Table S5), 60 against bull 2 (46 more and 14 less abundant, Table S6) and 47 against bull 3 (28 more and 19 less abundant, Table S7). The comparison between bull 2 and bull 3 revealed 40 DA proteins (18 more and 22 less abundant, Table S8), while in the other comparisons, no DA proteins were found (Tables S9 and S10). Across all individual pairwise comparisons with bull 4, a total of 17 proteins were shared, of which three proteins and 14 proteins were more and less abundant in bull 4, respectively (Figs 3B and S3). GSEA against Gene Ontology (GO) terms and pathways from the Kyoto Encyclopedia of Genes and Genomes (KEGG) in the comparison of bull 4 vs the other bulls showed three enriched gene sets with a positive enrichment score (organelle inner membrane, mitochondrial protein complex and oxidoreductase complex). When the high polyspermy bull (high polyspermy >50%, bull 1) and the low polyspermy control group (bulls 2, 3, and 4) were compared, no DA proteins were identified at the level of the sperm (Fig. S2, Table S11). GSEA in this comparison showed a total of 27 significantly enriched gene sets, 20 of which with a negative enrichment score and seven of which with a positive enrichment score. Negatively enriched gene sets were mainly associated with mitochondrial function and oxidative phosphorylation, whereas positively enriched sets were mainly related to protein synthesis and turnover and cytoskeleton-associated proteins (Fig. 4).

In total, 5,212 proteins were identified in the 2–4-cell embryo pools (Fig. S1). Individual pairwise comparisons did not show any DA proteins between pairs of bulls (Tables S12, S13, S14, S15, S16, S17). In the comparison between bull 4 vs bulls 1, 2 and 3, no DA proteins were found (Fig. S2, Table S18), while the comparison between the high polyspermy bull and the low polyspermy group showed one protein (serine incorporator 1/SERINC1) being significantly more and one protein (KH domain-containing RNA-binding protein QKI/QKI) being significantly less abundant in embryos fertilized with spermatozoa from the high polyspermy bull compared to the others (Figs S2 and S4A and Table S19). GSEA on the DA analysis results indicated reduced abundance of mTOR signaling-related proteins in bull 1 compared to the low polyspermy bulls (Fig. S4B).

**Figure 5:**
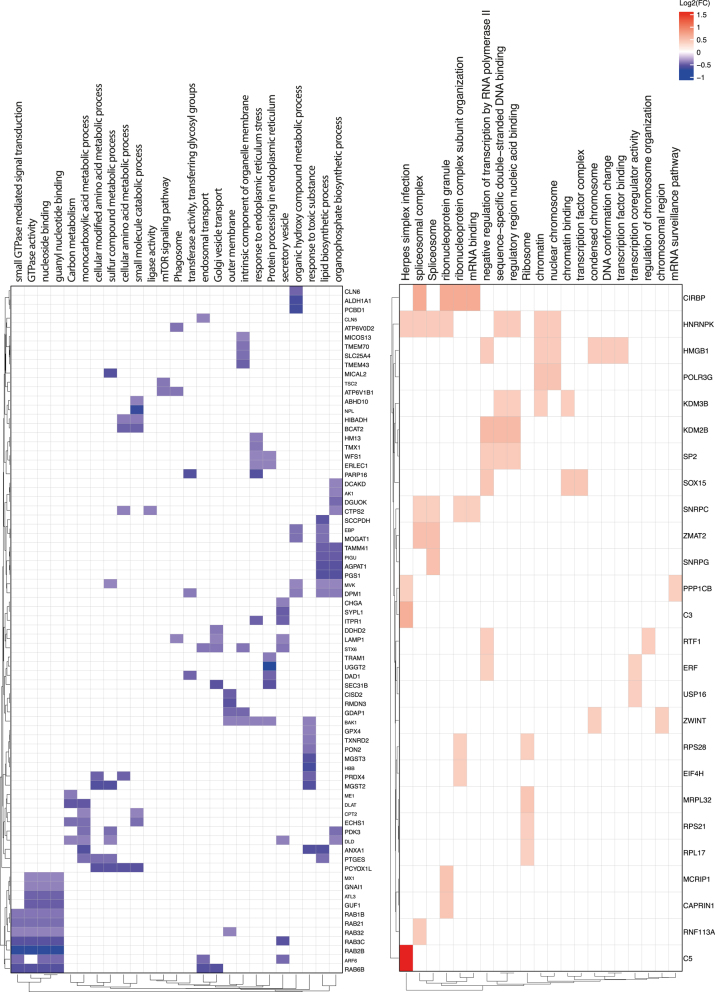
Gene Set Enrichment Analysis (GSEA) of the blastocyst from bull 4 compared to bulls 1, 2 and 3. The figure presents GSEA results using heat maps, including the list of genes (right side of each heat map) involved in the different pathways (on top of the heat map). The left side shows heat maps of down-regulated pathways in bull 4 (purple) while the right side shows heat maps of up-regulated pathways (red). Only proteins with an absolute log_2_ fold change of 1.3 or greater were plotted.

**Figure 6:**
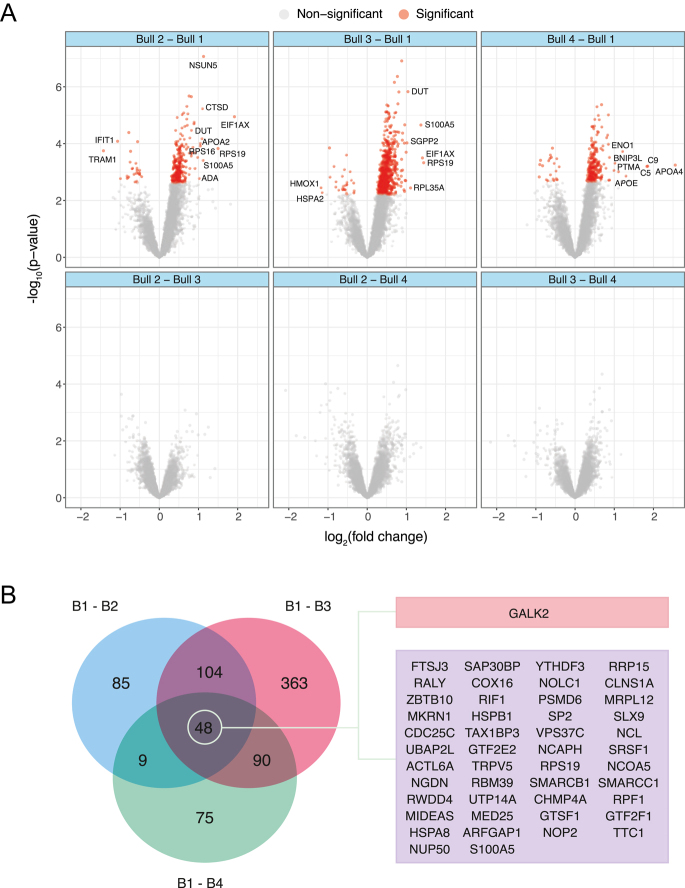
Comparative proteomics analysis of blastocyst from different bulls. Volcano plots showing differentially abundant (DA) proteins (adjusted *P*-value < 0.05) in pairwise comparisons between bulls. Each point represents a protein in volcano plots, with the X-axis denoting log_2_ fold change and the Y-axis denoting −log_10_ *P*-value. Red points represent differently abundant (DA) proteins (adjusted *P* value < 0.05). (B) Venn diagram illustrating DA proteins in comparisons against bull 1, with the center showing proteins common across all comparisons. Genes coding for down-regulated proteins are highlighted in purple. Genes coding for upregulated proteins are highlighted in red, and those coding for downregulated proteins are in purple.

**Figure 7:**
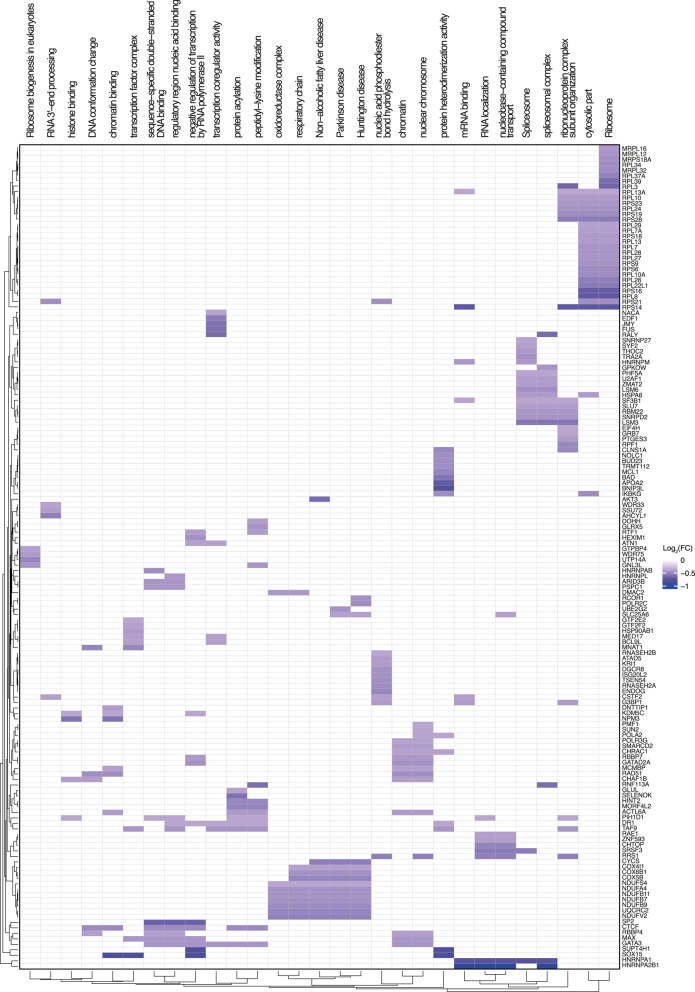
Gene Set Enrichment Analysis (GSEA) of the blastocyst from bull 1 compared to bulls 2, 3 and 4. The figure presents GSEA results using heat maps, including the list of genes (right side of the heat map) involved in the different pathways (on top of the heat map). Only proteins with an absolute log_2_ fold change of 1.3 or greater were plotted.

In total, 5,437 proteins were identified in the blastocyst (Fig. S1). No DA proteins were found in the comparison of bull 4 against the others (Fig. S2, Tables S20, S21, S22, S23). However, the GSEA in this comparison showed 25 significantly enriched gene sets with a negative score, mostly related to GTPase activity, binding and biosynthesis of nucleotides and lipid biosynthesis, and 21 with a positive score, mainly associated with chromosomes and transcription (Fig. 5). In the comparison between the high polyspermy bull and the low polyspermy group, 1,240 proteins were found to be DA (Fig. S2, Table S24). Specifically, 626 proteins were DA between bull 1 and bull 3 (601 less and 25 more abundant, Table S25), 250 proteins against bull 2 (226 less and 24 more abundant, Table S26) and 223 proteins against bull 4 (203 less and 20 more abundant, Table S27) (Fig. 6A). Across all individual pairwise comparisons with bull 1, a total of 48 proteins were shared, of which one protein and 47 proteins were more and less abundant in bull 1, respectively (Figs 6B and S5). The GSEA in the comparison of bull 1 and low polyspermy bulls showed that 29 signaling pathway-associated gene sets were enriched, all of which were downregulated. These gene sets were mainly associated with oxidative phosphorylation, chromatin organization, transcription, protein translation and posttranslational modifications (Fig. 7).

## Discussion

This study confirms an important bull effect on IVF outcomes, including polyspermic fertilization, embryo development and blastocyst quality, and stresses the necessity of evaluating bulls for polyspermy levels before applying them for *in vitro* embryo production, since polyspermic bulls may produce blastocysts (De Coster *et al.* 2022). A novel finding was that proteomic analysis of sperm, 2–4-cell embryos and blastocysts further uncovered subtle differences between bulls, with more pronounced variations observed in blastocysts.

Sperm motility kinematic parameters, including straightness (STR), wobble and the average amplitude of lateral head displacement (ALH), were different among bulls. The bull with the lowest penetration and cleavage rates exhibited the highest STR and the lowest ALH, which in bulls have been reported to be respectively negatively and positively correlated with cleavage and morula rates (Hasbi *et al.* 2024). Amplitude of lateral head displacement is associated with hyperactivation and acrosome reaction in the sperm, which are necessary to penetrate the zona pellucida, possibly explaining the low penetration rates of this bull (Yanagimachi 1994).

The bull with the highest penetration rate exhibited the highest cleavage rate, but also the highest polyspermy and the lowest IVF efficiency. Despite this, its blastocyst rate was almost equal to that of the bull with the lowest polyspermy rate. These results confirm our previous study with high blastocyst rates in a polyspermic bull (Fernández-Montoro *et al.* 2024), but differ from studies on pigs (Nguyen *et al.* 2020) and sheep (Anzalone *et al.* 2021), in which increased polyspermy rates tended to reduce blastocyst rates. Blastocysts derived from the high polyspermy bull seem to be of lower quality, as they tended to have lower ICM and ICM/TCN ratios. Our findings align with studies in pigs showing that embryos from high polyspermy conditions present significantly lower ICM counts, with or without differences in TCN (Han *et al.* 1999, Koo *et al.* 2005). This reduction might explain a self-correction mechanism from the embryo, in which aneuploid blastomeres in the ICM derived from polyspermic fertilization are more prone to trigger cell arrest or apoptosis (Santos *et al.* 2010, Bolton *et al.* 2016).

At the level of the sperm, the bull with the lowest penetration and cleavage rates, yet the highest blastocyst quality (bull 4) exhibited the greatest differences in the number of DA proteins. Among low abundance proteins were seminal plasmin (PYY2) and seminal ribonuclease (SRN), the first one being involved in acrosome exocytosis and the second one in sperm capacitation, immunosuppression, antioxidant function and catalytic activity (Clark *et al.* 1993, Kim *et al.* 1995). In addition, SRN relates to high viability of frozen-thawed bull sperm and high bull fertility (Viana *et al.* 2018, Gomes *et al.* 2020), hence explaining the low penetration rates observed in this bull. TIMP metallopeptidase inhibitor 2 (TIMP2) and epididymal sperm-binding protein 1 (ELSPBP1) levels have been reported to be respectively negatively and positively correlated to DNA fragmentation in humans (Belardin *et al.* 2019, 2023). In bull 4, TIMP2 showed lower abundance while ELSPBP1 was more abundant. Although we did not assess it, an increase in DNA fragmentation levels might explain the low cleavage rates observed in this bull. It has been demonstrated that bovine sperm DNA fragmentation reduces cleavage rates without affecting the blastocyst rate or the number of blastomeres (Simões *et al.* 2013), coinciding with the embryo development and quality outcomes of bull 4.

Although no DA proteins were found among the high and low polyspermy bulls, GSEA showed seven overrepresented pathways in the high polyspermy bull. Among them, we found ‘response to abiotic stimulus’. We hypothesize that the upregulation of this pathway might relate to an increased sensitivity of the sperm to respond to stimuli from the environment, such as capacitation-stimulating components of the media (i.e., heparin). Among the proteins involved in this pathway, we found upregulated junction plakoglobin (JUP), a protein identified in capacitated bovine sperm (Rajamanickam *et al.* 2019). A more easily triggered acrosomal reaction could explain the high penetration rates in this bull. In contrast, pathways related to mitochondrial function were downregulated in the high polyspermy bull and upregulated in the bull with the best blastocyst quality. A comparative study of the sperm proteome of bulls with contrasting field fertility identified those same pathways as the most significantly affected in low-fertile bulls (Saraf *et al.* 2022).

The proteome of 2–4-cell embryos was not different among bulls. That is in disagreement with previous studies that found differences in the transcriptome of 2–4-cell embryos. Nevertheless, these studies compared sires that greatly differ in their fertility status (Gross *et al.* 2019, Lockhart *et al.* 2023), while the bulls used in our research exhibited similar field fertility and blastocyst rates. In the comparison between the high polyspermy bull and the others, only the mammalian target of rapamycin (mTOR) pathway was downregulated. In mice, the confrontation of cells with different fitness levels (i.e., tetraploid and wild-type cells) led to an inhibition of mTOR signaling in the less-fit cell type, causing its elimination (Bowling *et al.* 2018). Although it is yet to confirm, downregulation of this pathway in embryos from bull 1 might reflect an increased incidence of karyotypic abnormalities derived from polyspermic fertilization, which led to high apoptosis in those cells.

The greatest impact of the bull on the proteome was observed at the blastocyst stage. A great number of DA proteins downregulated in the embryos resulting from the high polyspermy bull were associated with oxidative phosphorylation. Metabolic demands increase between morula compaction and blastocyst formation, with about 86% of ATP resulting from aerobic respiration (De Souza *et al.* 2015). The mitochondrial respiration signature correlated to embryo quality in cattle (Kurzella *et al.* 2023). Proteins associated with transcription and translation were also found downregulated in the high polyspermy bull. These pathways were reported to be downregulated in IVP embryos as opposed to those developed *in vivo*, which are generally recognized to be of higher quality (Corcoran *et al.* 2006). The lack of the necessary machinery to produce enough biological mass and allow their expansion at the time of implantation together with a lower mitochondrial function might indicate a compromised developmental potential of the blastocyst from the high polyspermy bull.

Previous research has demonstrated the impact of the bull on the sperm proteome or embryo transcriptome (Peddinti *et al.* 2008, Lockhart *et al.* 2023). However, these studies did not analyze simultaneously sperm and embryos from the same bull. In our study, the specific DA proteins and ontologies in the comparison of the sperm proteomes of different bulls differed from those found in the embryonic proteomes. Only in the high polyspermy bull, both sperm and blastocysts indicated downregulation in pathways related to mitochondria. Still, no other proteins or pathways were commonly regulated between the three analyzed stages. For the bull with the highest embryo quality, while mitochondrial-related pathways were upregulated in the sperm, this pattern did not extend to the embryos. In addition, we identified five proteins indicative of low-quality sperm in the bull with the best embryo quality, suggesting a divergence between the proteomic profiles of sperm and embryos. This could be explained by the different proteomic requirements of sperm and embryos, while the sperm cell is highly specialized for motility and delivery of the genome to the oocyte, early embryos undergo rapid mitotic divisions and differentiation (Miyazaki *et al.* 2023).

While our study provides valuable insights into the complex interplay between bull traits, fertilization and embryonic outcomes, it is crucial to acknowledge its inherent limitations. First, the sample size of bulls included in our study was limited, with only one bull exhibiting polyspermy, restricting the generalizability of our findings to a broader population of bulls. Moreover, determination of polyspermic fertilization (by fixing and staining) was performed on a different subset of presumed zygotes than those used for embryo development. Even though the same ejaculates were used, conclusions are therefore indirect. Our proteomic analysis also relied on randomly selected samples. Consequently, we cannot definitively confirm the occurrence of polyspermic fertilization, potentially confounding our comparison between embryos resulting from polyspermic fertilization and those with normal fertilization. Yet, the overall occurrence of polyspermic embryos is expected to be higher due to the empirically observed elevated polyspermy rate in bull 1. Future investigations with larger sample sizes and specifically selected embryos based on cleavage patterns such as multipolar division, which has been correlated with polyspermic fertilization, or using live-imaging of zygotes (Plachta *et al.* 2011, De Coster *et al.* 2022, Bissiere *et al.* 2023), could corroborate and expand our findings.

In conclusion, we demonstrated a clear bull effect from sperm to blastocyst, which was reflected in our proteome data. Fertile bulls with similar sperm characteristics can yield varying IVF results, with high polyspermy correlating with diminished IVF efficiency but not necessarily with reduced blastocyst rates. The sperm proteome primarily reflected bull-specific reduced penetration and cleavage rates, but not polyspermic fertilization, while the proteome signature at the blastocyst level distinguished the bull with the highest polyspermy rates. Blastocysts resulting from the high polyspermy bull exhibited lower quality, as indicated by imbalances in pathways required for embryo development and implantation. These results provide insightful information about the intricate interactions between characteristics of sperm, fertilization outcomes, embryo development and quality of bulls with different IVF efficiency, and highlight the importance of selecting bulls carefully by testing them in a separate experiment for penetration and polyspermy rate, before allowing them to be used for *in vitro* embryo production.

## Supplementary materials





















## Declaration of interest

The authors declare that there is no conflict of interest that could be perceived as prejudicing the impartiality of the work reported.

## Funding

This research was supported by the European Union’s Horizon 2020 research and innovation program under the Marie Skłodowska-Curie grant agreement No 860960. KG acknowledges support from The Research Foundation – Flanders (FWO), iBOF project, project number 860960 IBOF-23-005.

## Author contribution statement

AF, EA, KCP, MB, TDC, KS and AVS conceptualized and designed the study. AF and TDC conducted the *in vitro* experiments. DAV and MH helped in the experimental work. AF and DAV carried out the statistical analysis. EA and MB performed the proteomics sample preparation, LC-MS/MS and the proteomics data analysis. AF, EA, MB, DAV and KS interpreted the results and wrote the manuscript. AVS, KCP and KG edited and approved the final version. All authors have read and agreed to the published version of the manuscript.

## Data availability

The mass spectrometry proteomics data have been deposited to the ProteomeXchange Consortium via the PRIDE partner repository with the dataset identifier PXD054561.
