# Supplementary material for: Decoding bull fertility in vitro: a proteomics exploration from sperm to blastocyst
Source: Reproduction. 2025 Mar 19;169(4):e240296. doi: 10.1530/REP-24-0296 (PMC11949518; doi:10.1530/REP-24-0296)
Supplement: Supplementary file 1 [file tables_s1-s3.pdf]

Table S1. Dia-PASEF windows

Cycle Id	Start IM [1/K0]	End IM [1/K0]	Start Mass [m/z]	End Mass [m/z]
1	0.64	0.83	400	425
2	0.64	0.85	425	450
3	0.64	0.87	450	475
4	0.64	0.9	475	500
5	0.64	0.92	500	525
6	0.64	0.94	525	550
7	0.64	0.97	550	575
8	0.64	0.99	575	600
1	0.83	1.01	600	625
2	0.85	1.04	625	650
3	0.87	1.06	650	675
4	0.9	1.09	675	700
5	0.92	1.11	700	725
6	0.94	1.13	725	750
7	0.97	1.16	750	775
8	0.99	1.18	775	800
1	1.01	1.37	800	825
2	1.04	1.37	825	850
3	1.06	1.37	850	875
4	1.09	1.37	875	900
5	1.11	1.37	900	925
6	1.13	1.37	925	950
7	1.16	1.37	950	975
8	1.18	1.37	975	1000

Table S2. Assessment of fertilization parameters in zygotes generated from oocytes fertilized with sperm from four different bulls.

Treatment	No. of presumed zygotes	Penetration (%)[1A]	IVF efficiency (%)[1B]	No. of penetrated zygotes	Polyspermy (%)[1C]	PN decondensation[1D]	PN apposition[1D]	No. of polyspermic zygotes	No. of sperm[2E]
Bull 1	261	93.4 ± 2.0[a]	40.9 ± 3.5[a]	240	56.1 ± 4.8[a]	83.9 ± 1.4[a]	55.9 ± 2.8	133	3.1 ± 1.8
Bull 2	225	73.6 ± 5.2[b]	52.2 ± 3.8[ab]	160	24.9 ± 4.4[b]	90.4 ± 1.7[bc]	57.1 ± 3.5	41	2.6 ± 1.1
Bull 3	272	79.6 ± 4.3[b]	60 ± 3.4[b]	203	19.3 ± 3.6[b]	94.3 1.5[b]	60.3 ± 3.1	38	2.5 ± 1.2
Bull 4	121	66 ± 7.3[b]	57 ± 5.0[b]	92	19.4 ± 4.8[b]	85.5 ± 2.3[ac]	54.5 ± 4.6	22	2.5 ± 1

[A] Penetration rate is expressed as the number of zygotes penetrated at least by one sperm cell over the presumed zygotes.

[B] IVF efficiency is estimated as the number of monospermic zygotes out of the number of presumed zygotes.

[C] Polyspermy rate is calculated as the number of zygotes penetrated by more than one sperm cell over the number of penetrated zygotes.

[D] PN (pronuclei) decondensation and apposition are estimated as the number of decondensed and apposed pronuclei from the total number of pronuclei in the zygote.

[E] Number of sperm is expressed as the number of sperm tails counted in polyspermic zygotes.

Different superscripts (a, b and c) per column represent statistical differences ($p < 0.05$) among groups. Results are expressed as least-square means ± SE[1] or means ± standard deviation[2]

Table S3. Correlation coefficients between sperm traits, fertilization, and developmental parameters (*n* = 26).

Variable 1	Variable 2	Pearson correlation value [A]	p-value
Total motility (%)	Penetration rate (%)	0.4016	0.0420
	IVF efficiency (%)	-0.0725	0.7248
	Polyspermy rate (%)	0.3174	0.1141
	Average sperm cell count	0.3792	0.0561
	Decondensation rate (%)	-0.2401	0.2374
	Apposition rate (%)	0.3414	0.0879
	Cleavage rate (%)	0.1303	0.5258
	Blastocyst rate day 7 (%)	0.4304	0.0282
	Blastocyst rate day 8 (%)	0.2341	0.2496
Progressive motility (%)	Penetration rate (%)	0.4208	0.0323
	IVF efficiency (%)	-0.1251	0.5425
	Polyspermy rate (%)	0.3714	0.0618
	Average sperm cell count	0.4132	0.0359
	Decondensation rate (%)	-0.3208	0.1101
	Apposition rate (%)	0.4041	0.0406
	Cleavage rate (%)	0.3044	0.1306
	Blastocyst rate day 7 (%)	0.5053	0.0085
	Blastocyst rate day 8 (%)	0.3208	0.1100
Curvilinear velocity (VCL: µm/s)	Penetration rate (%)	0.2843	0.1593
	IVF efficiency (%)	-0.0931	0.6509
	Polyspermy rate (%)	0.2928	0.1466
	Average sperm cell count	0.2523	0.2137
	Decondensation rate (%)	-0.1793	0.3809
	Apposition rate (%)	0.2840	0.1597
	Cleavage rate (%)	0.4109	0.0371
	Blastocyst rate day 7 (%)	0.4806	0.0130
	Blastocyst rate day 8 (%)	0.4253	0.0303

Variable 1	Variable 2	Pearson correlation value [A]	p-value
Straight-line velocity (VSL: µm/s)	Penetration rate (%)	0.1723	0.4001
	IVF efficiency (%)	0.0643	0.7549
	Polyspermy rate (%)	0.1082	0.5986
	Average sperm cell count	0.0521	0.8004
	Decondensation rate (%)	-0.3640	0.0676
	Apposition rate (%)	0.1792	0.3812
	Cleavage rate (%)	0.3995	0.0432
	Blastocyst rate day 7 (%)	0.3999	0.0430
	Blastocyst rate day 8 (%)	0.3817	0.0544
Average path velocity (VAP: µm/s)	Penetration rate (%)	0.1681	0.4118
	IVF efficiency (%)	0.0363	0.8604
	Polyspermy rate (%)	0.1159	0.5730
	Average sperm cell count	0.0614	0.7656
	Decondensation rate (%)	-0.3613	0.0698
	Apposition rate (%)	0.1947	0.3405
	Cleavage rate (%)	0.3845	0.0524
	Blastocyst rate day 7 (%)	0.4183	0.0335
	Blastocyst rate day 8 (%)	0.3669	0.0652
Linearity (LIN (VSL/VCL): %)	Penetration rate (%)	0.0366	0.8591
	IVF efficiency (%)	0.1713	0.4029
	Polyspermy rate (%)	-0.0645	0.7541
	Average sperm cell count	-0.1161	0.5723
	Decondensation rate (%)	-0.4187	0.0333
	Apposition rate (%)	0.0572	0.7814
	Cleavage rate (%)	0.2498	0.2184
	Blastocyst rate day 7 (%)	0.2216	0.2767
	Blastocyst rate day 8 (%)	0.2373	0.2430
Straightness (STR (VSL/VAP,): %)	Penetration rate (%)	0.1686	0.4104
	IVF efficiency (%)	0.0944	0.6464

Variable 1	Variable 2	Pearson correlation value [A]	p-value
Straightness (STR (VSL/VAP,): %)	Polyspermy rate (%)	0.1067	0.6038
	Average sperm cell count	0.0509	0.8050
	Decondensation rate (%)	-0.2423	0.2330
	Apposition rate (%)	0.1076	0.6009
	Cleavage rate (%)	0.3619	0.0693
	Blastocyst rate day 7 (%)	0.2981	0.1391
	Blastocyst rate day 8 (%)	0.3642	0.0673
Wobble (WOB (VAP/VCL): %)	Penetration rate (%)	-0.0407	0.8437
	IVF efficiency (%)	0.1890	0.3552
	Polyspermy rate (%)	-0.1485	0.4690
	Average sperm cell count	-0.1962	0.3367
	Decondensation rate (%)	-0.4320	0.0275
	Apposition rate (%)	0.0285	0.8899
	Cleavage rate (%)	0.1698	0.4070
	Blastocyst rate day 7 (%)	0.1689	0.4095
	Blastocyst rate day 8 (%)	0.1556	0.4479
Lateral head displacement (ALH: µm)	Penetration rate (%)	0.3139	0.1183
	IVF efficiency (%)	-0.1153	0.5749
	Polyspermy rate (%)	0.3146	0.1176
	Average sperm cell count	0.3471	0.0823
	Decondensation rate (%)	0.1866	0.3615
	Apposition rate (%)	0.3747	0.0593
	Cleavage rate (%)	0.2586	0.2021
	Blastocyst rate day 7 (%)	0.3426	0.0867
	Blastocyst rate day 8 (%)	0.3125	0.1201
Beat cross frequency (BCF: number per second)	Penetration rate (%)	0.0100	0.9615
	IVF efficiency (%)	0.0485	0.8141
	Polyspermy rate (%)	0.0141	0.9456
	Average sperm cell count	-0.0588	0.7754

Variable 1	Variable 2	Pearson correlation value [A]	p-value
Beat cross frequency (BCF: number per second)	Decondensation rate (%)	0.0682	0.7408
	Apposition rate (%)	-0.0087	0.9663
	Cleavage rate (%)	0.0177	0.9316
	Blastocyst rate day 7 (%)	0.1514	0.4602
	Blastocyst rate day 8 (%)	0.3101	0.1232
Penetration rate (%)	Cleavage rate (%)	0.4556	0.0194
	Blastocyst rate day 7 (%)	0.6040	0.0011
	Blastocyst rate day 8 (%)	0.4589	0.0184
IVF efficiency (%)	Cleavage rate (%)	-0.2803	0.1654
	Blastocyst rate day 7 (%)	-0.1092	0.5954
	Blastocyst rate day 8 (%)	0.0837	0.6844
Polyspermy rate (%)	Cleavage rate (%)	0.5460	0.0039
	Blastocyst rate day 7 (%)	0.5265	0.0057
	Blastocyst rate day 8 (%)	0.2934	0.1457
Penetration rate (%)	IVF efficiency (%)	0.0723	0.7256
	Polyspermy rate (%)	0.7176	0.0000

[A] Significant correlation $p < 0.05$.
