# Supplementary figures and images for: Decoding bull fertility in vitro: a proteomics exploration from sperm to blastocyst
Source: Reproduction. 2025 Mar 19;169(4):e240296. doi: 10.1530/REP-24-0296 (PMC11949518; doi:10.1530/REP-24-0296)

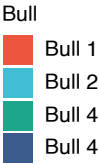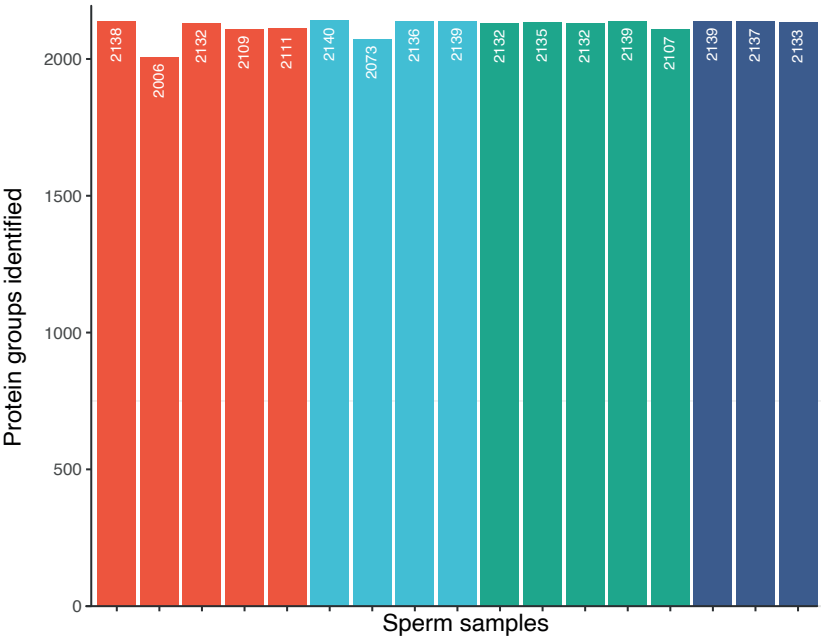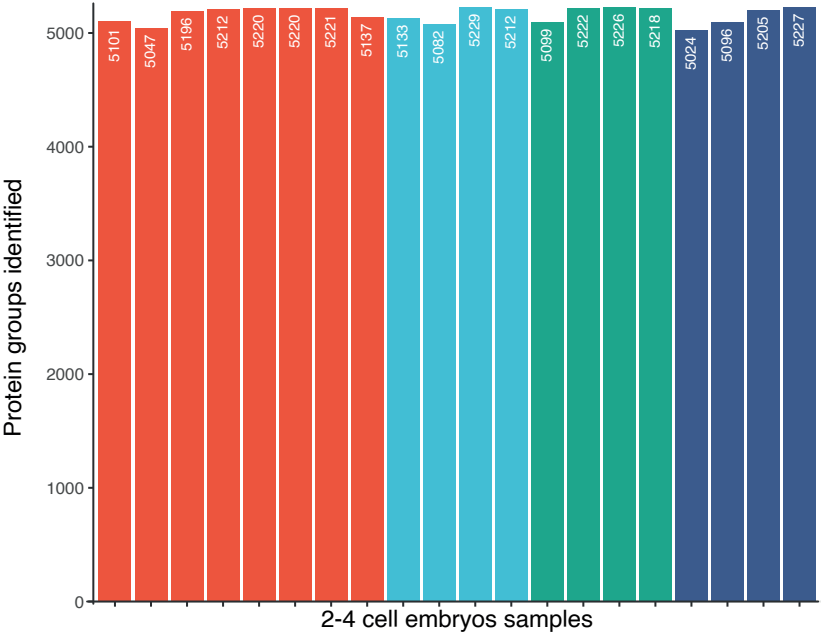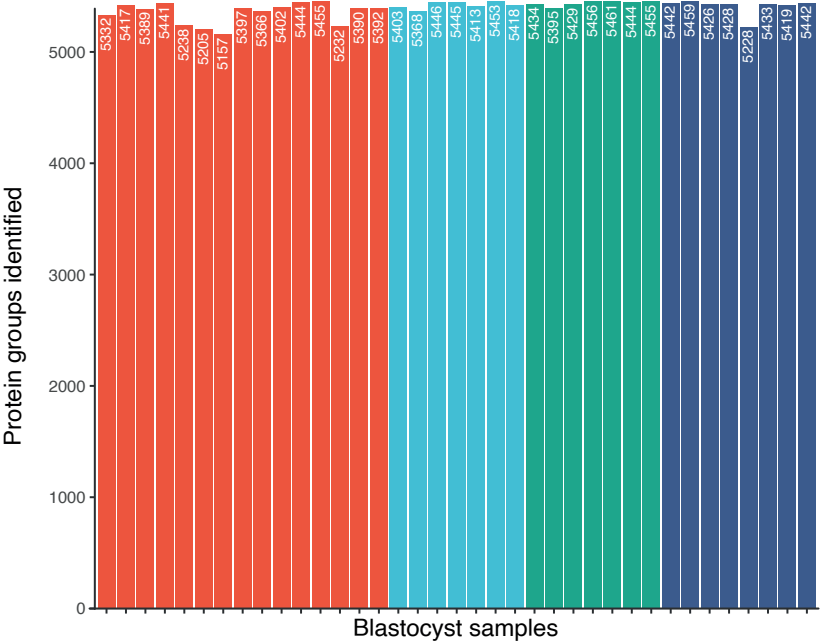

Supplement: Supplementary file 2 [file figure_s1.pdf]

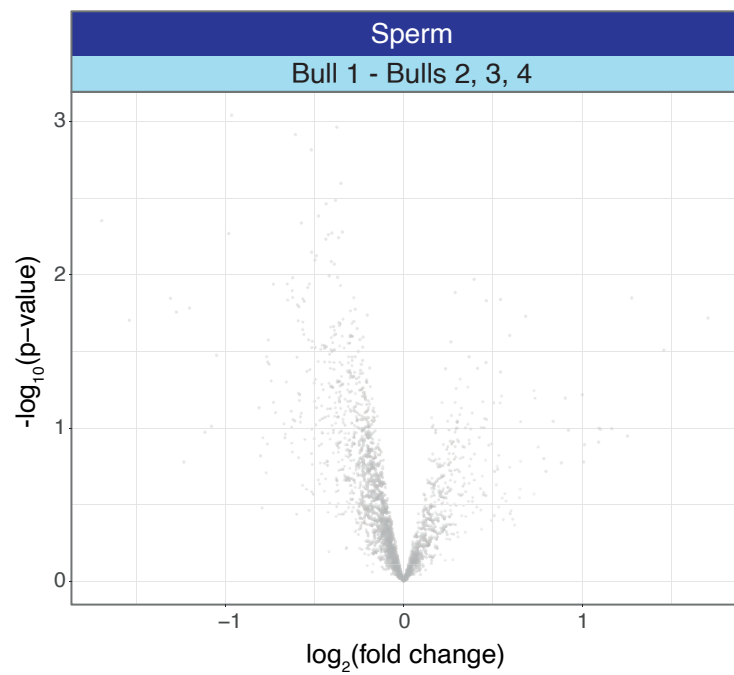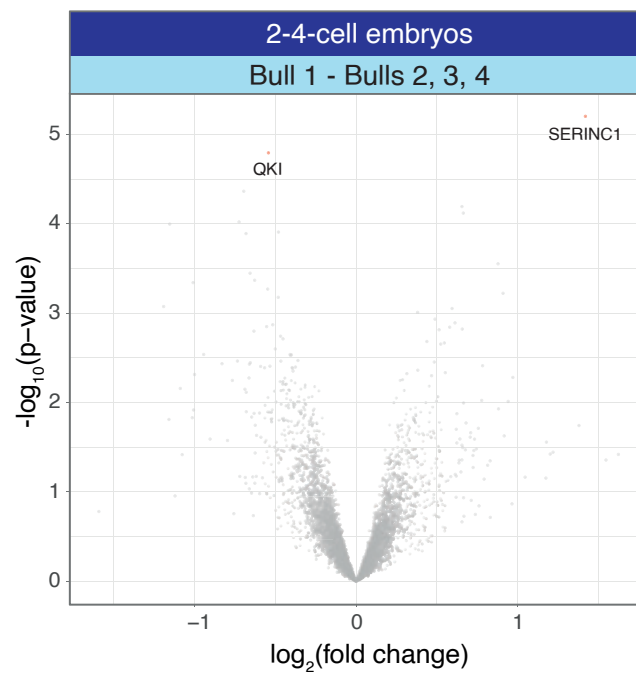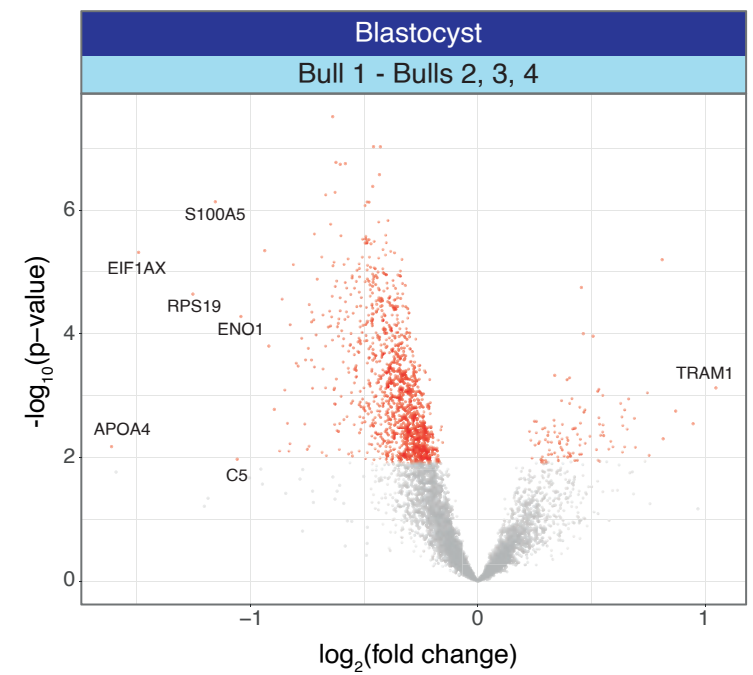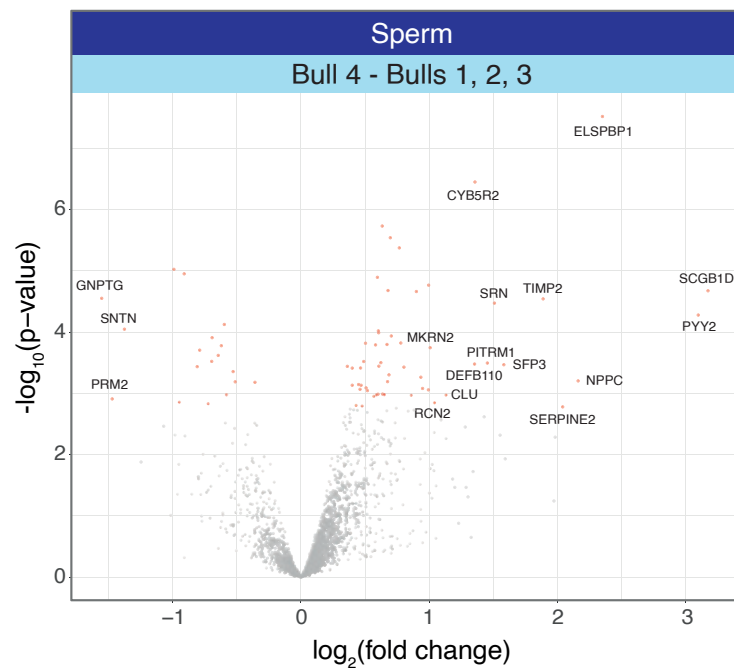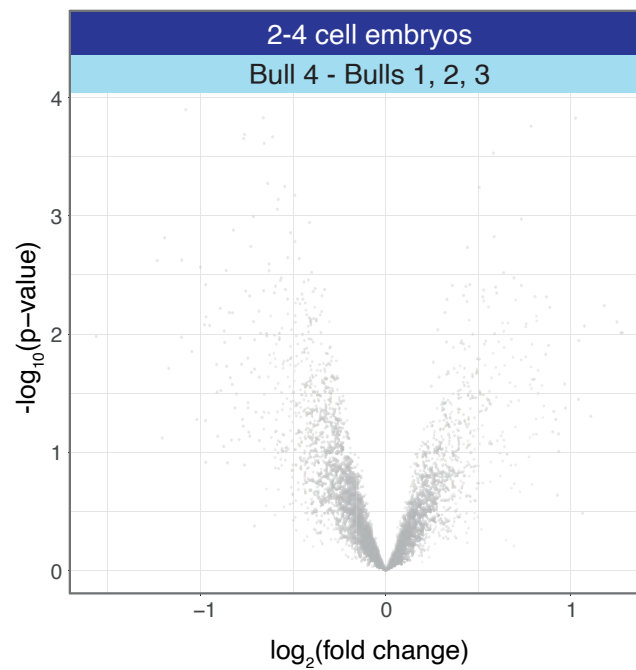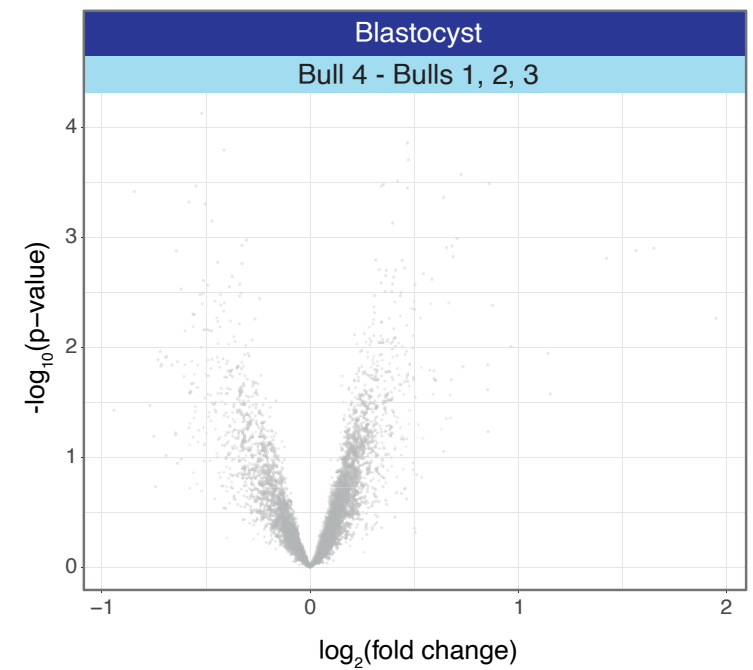

Supplement: Supplementary file 3 [file figure_s2.pdf]

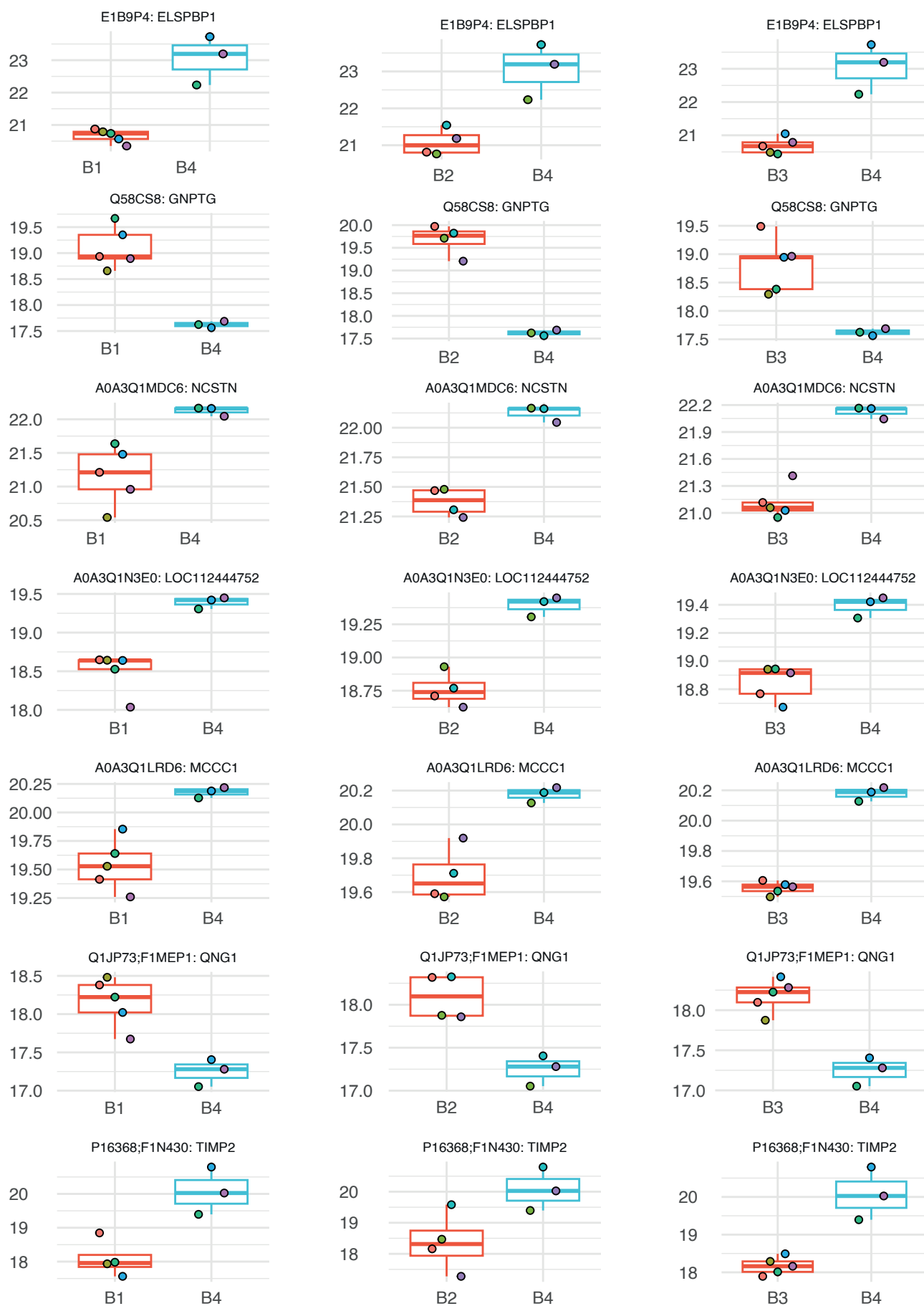

B: bull      replicate      R1      R2      R3      R4      R5

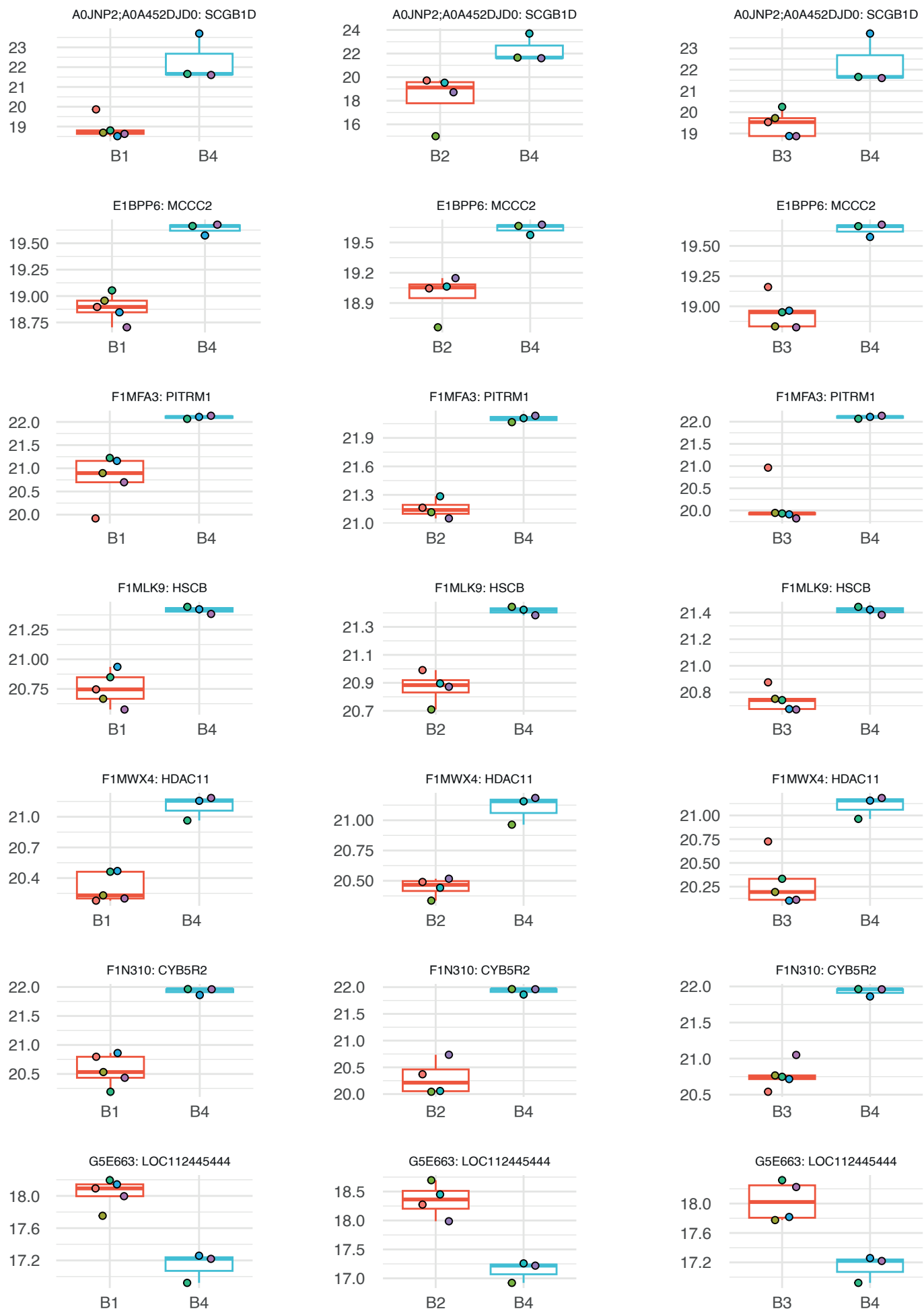

B: bull      replicate      ● R1      ● R2      ● R3      ● R4      ● R5

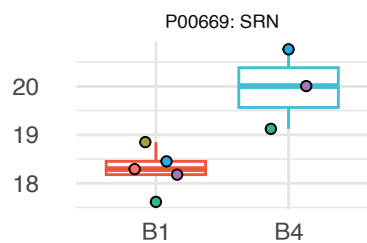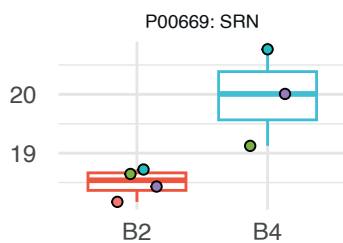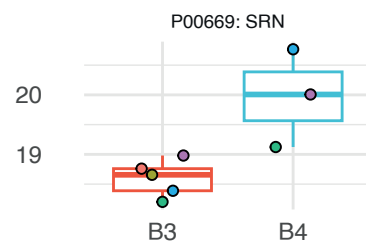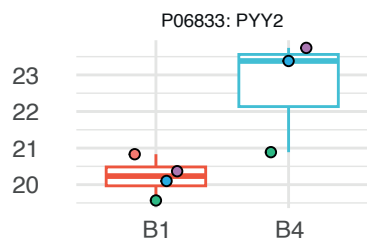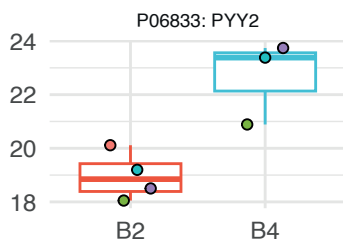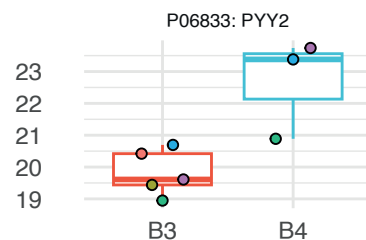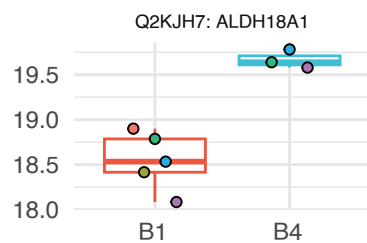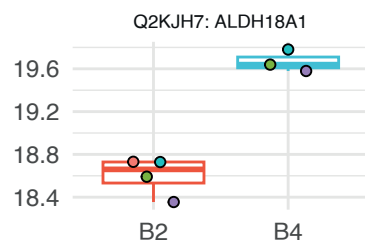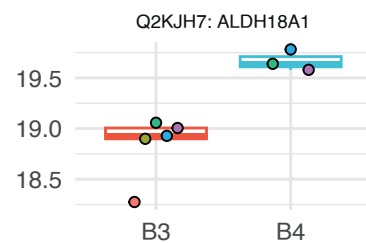

B: bull      replicate      ● R1   ● R2   ● R3   ● R4   ● R5

Supplement: Supplementary file 5 [file figure_s3.pdf]

A

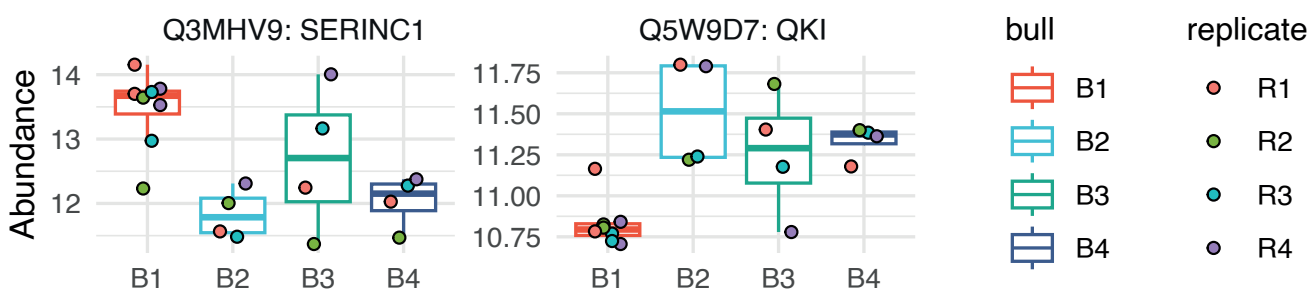

B

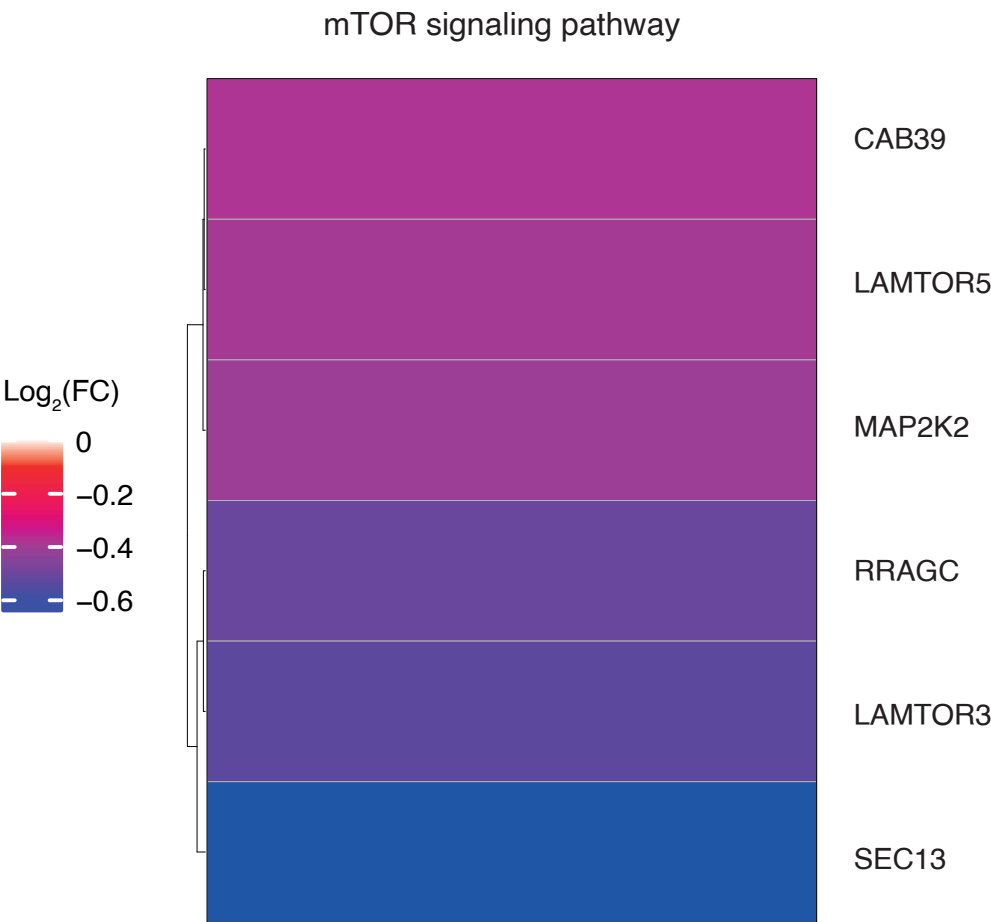

Supplement: Supplementary file 7 [file figure_s4.pdf]
