# Supplementary material for: Decoding bull fertility in vitro: a proteomics exploration from sperm to blastocyst
Source: Reproduction. 2025 Mar 19;169(4):e240296. doi: 10.1530/REP-24-0296 (PMC11949518; doi:10.1530/REP-24-0296)
Supplement: Supplementary file 9 [file figure_s5.pdf]

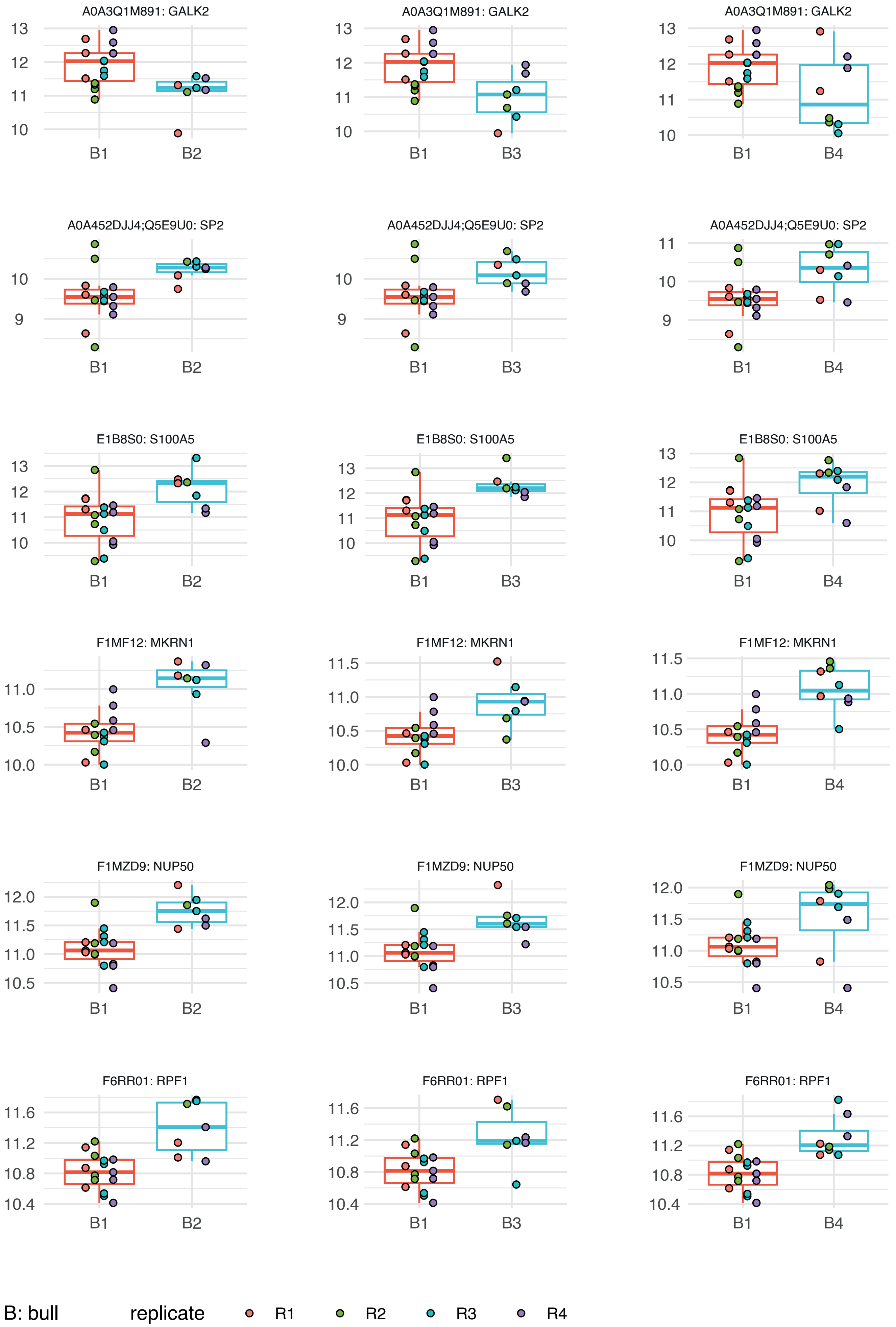
A0A3Q1M891: GALK2
A0A452DJJ4;Q5E9U0: SP2
E1B8S0: S100A5
F1MF12: MKRN1
F1MZD9: NUP50
F6RR01: RPF1
B1
B2
B3
B4
B: bull
replicate
R1
R2
R3
R4

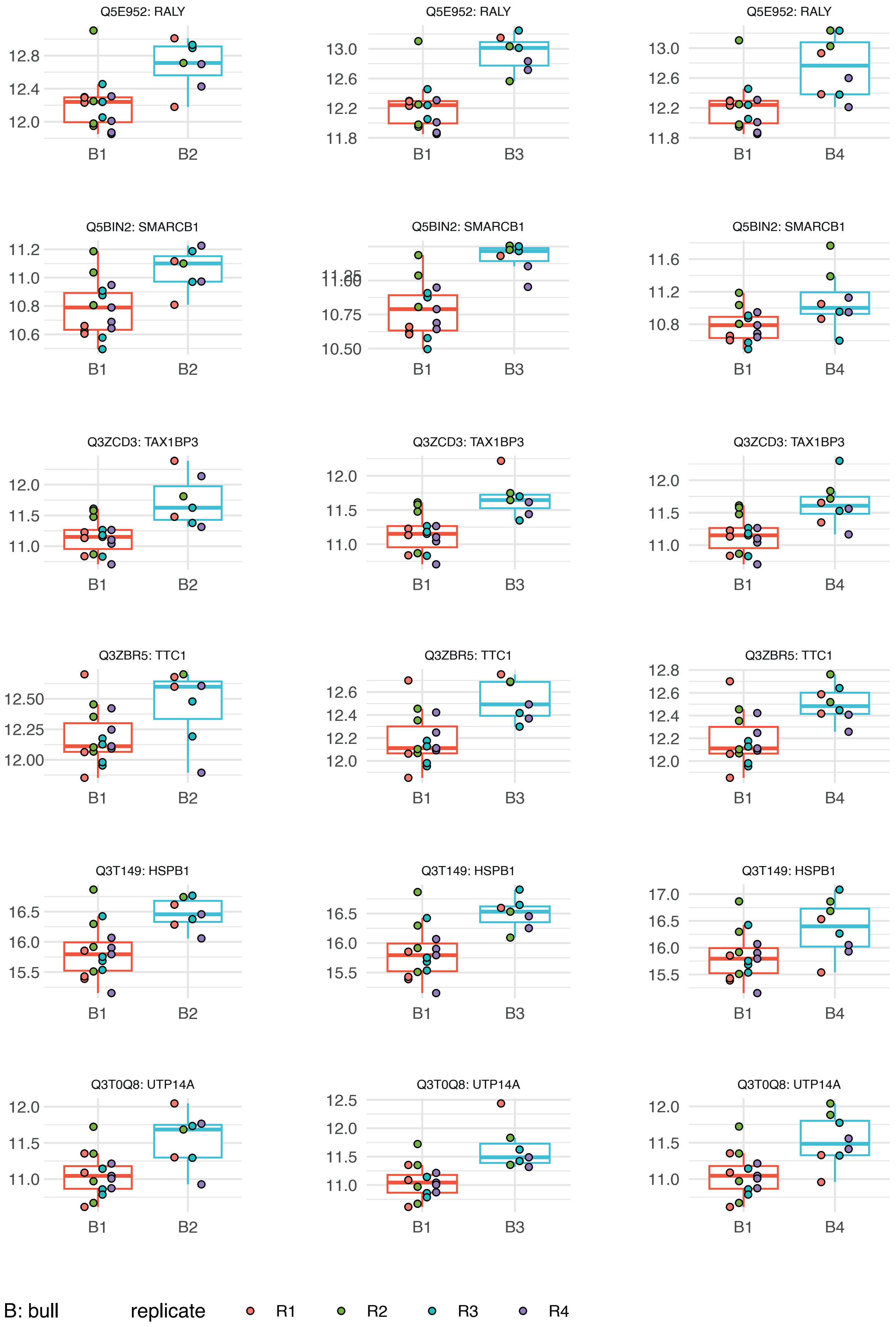
Q5E952: RALY
B1
B2
Q5E952: RALY
B1
B3
Q5E952: RALY
B1
B4
Q5BIN2: SMARCB1
B1
B2
Q5BIN2: SMARCB1
B1
B3
Q5BIN2: SMARCB1
B1
B4
Q3ZCD3: TAX1BP3
B1
B2
Q3ZCD3: TAX1BP3
B1
B3
Q3ZCD3: TAX1BP3
B1
B4
Q3ZBR5: TTC1
B1
B2
Q3ZBR5: TTC1
B1
B3
Q3ZBR5: TTC1
B1
B4
Q3T149: HSPB1
B1
B2
Q3T149: HSPB1
B1
B3
Q3T149: HSPB1
B1
B4
Q3T0Q8: UTP14A
B1
B2
Q3T0Q8: UTP14A
B1
B3
Q3T0Q8: UTP14A
B1
B4
B: bull
replicate
R1
R2
R3
R4

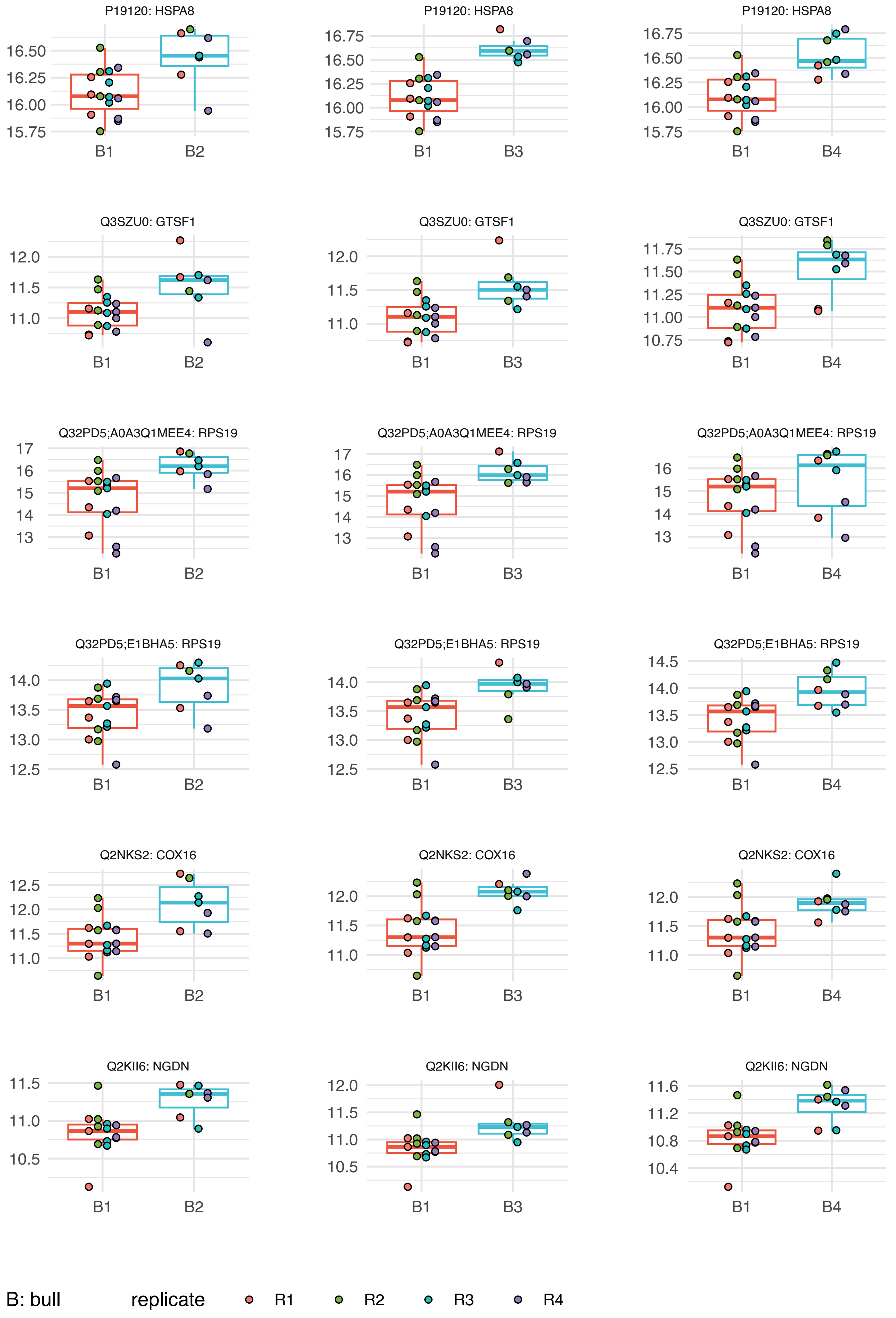
P19120: HSPA8
B1
B2
P19120: HSPA8
B1
B3
P19120: HSPA8
B1
B4
Q3SZU0: GTSF1
B1
B2
Q3SZU0: GTSF1
B1
B3
Q3SZU0: GTSF1
B1
B4
Q32PD5;A0A3Q1MEE4: RPS19
B1
B2
Q32PD5;A0A3Q1MEE4: RPS19
B1
B3
Q32PD5;A0A3Q1MEE4: RPS19
B1
B4
Q32PD5;E1BHA5: RPS19
B1
B2
Q32PD5;E1BHA5: RPS19
B1
B3
Q32PD5;E1BHA5: RPS19
B1
B4
Q2NKS2: COX16
B1
B2
Q2NKS2: COX16
B1
B3
Q2NKS2: COX16
B1
B4
Q2KII6: NGDN
B1
B2
Q2KII6: NGDN
B1
B3
Q2KII6: NGDN
B1
B4
B: bull
replicate
R1
R2
R3
R4

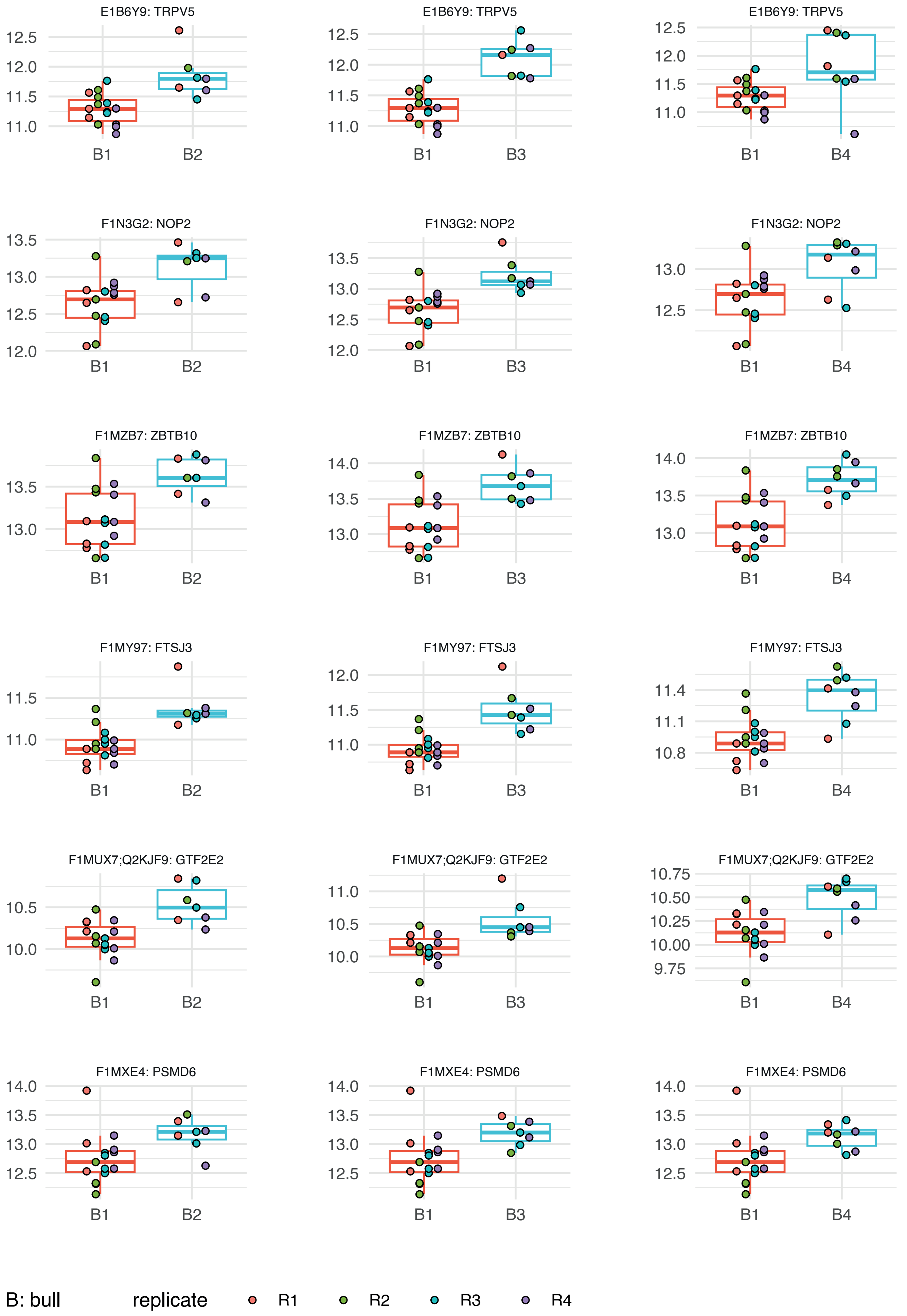
E1B6Y9: TRPV5
F1N3G2: NOP2
F1MZB7: ZBTB10
F1MY97: FTSJ3
F1MUX7;Q2KJF9: GTF2E2
F1MXE4: PSMD6
B1
B2
B3
B4
B: bull
replicate
R1
R2
R3
R4

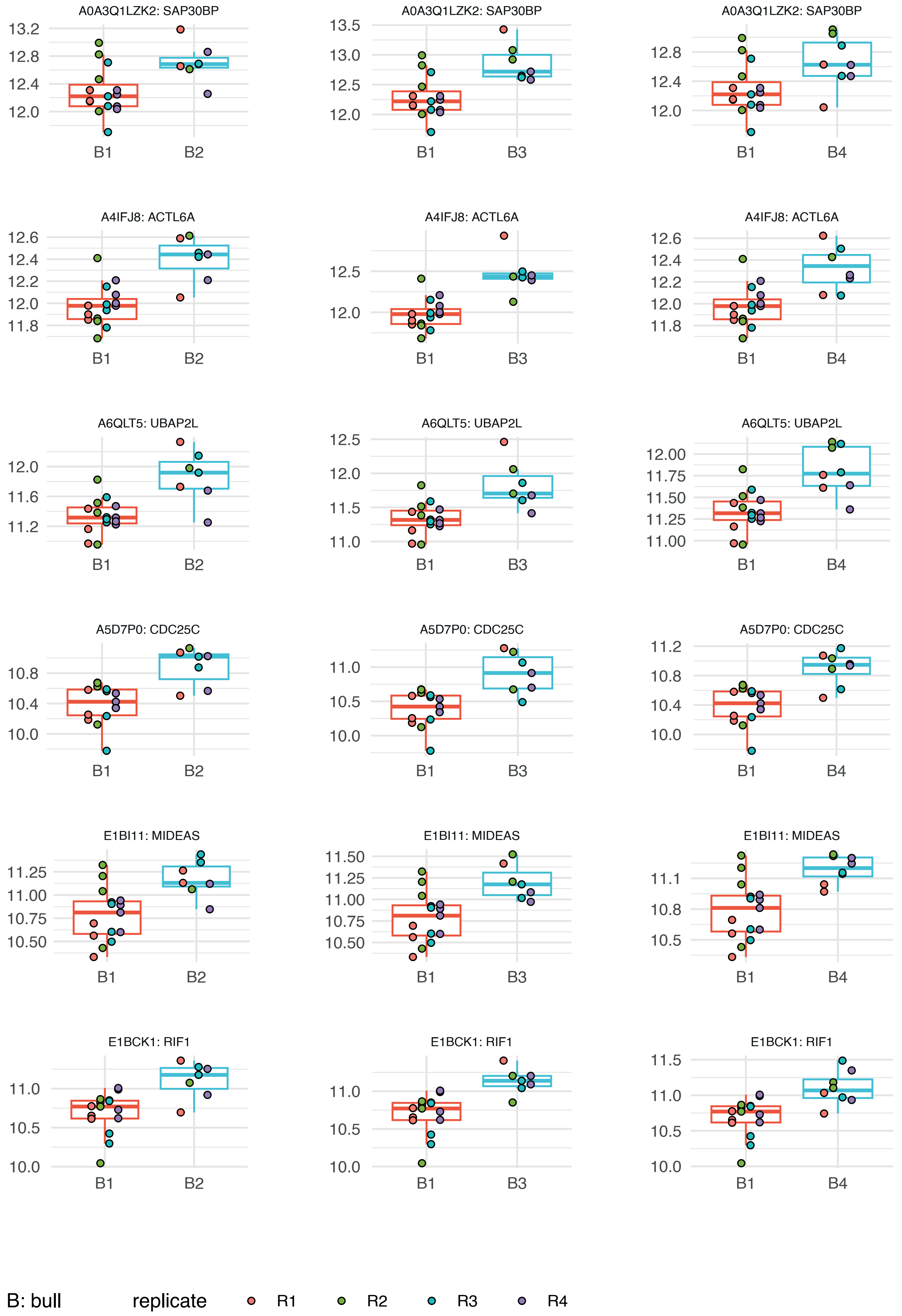
A0A3Q1LZK2: SAP30BP
13.2
12.8
12.4
12.0
B1
B2
A0A3Q1LZK2: SAP30BP
13.5
13.0
12.5
12.0
B1
B3
A0A3Q1LZK2: SAP30BP
12.8
12.4
12.0
B1
B4
A4IFJ8: ACTL6A
12.6
12.4
12.2
12.0
11.8
B1
B2
A4IFJ8: ACTL6A
12.5
12.0
B1
B3
A4IFJ8: ACTL6A
12.6
12.4
12.2
12.0
11.8
B1
B4
A6QLT5: UBAP2L
12.0
11.6
11.2
B1
B2
A6QLT5: UBAP2L
12.5
12.0
11.5
11.0
B1
B3
A6QLT5: UBAP2L
12.00
11.75
11.50
11.25
11.00
B1
B4
A5D7P0: CDC25C
10.8
10.4
10.0
B1
B2
A5D7P0: CDC25C
11.0
10.5
10.0
B1
B3
A5D7P0: CDC25C
11.2
10.8
10.4
10.0
B1
B4
E1BI11: MIDEAS
11.25
11.00
10.75
10.50
B1
B2
E1BI11: MIDEAS
11.50
11.25
11.00
10.75
10.50
B1
B3
E1BI11: MIDEAS
11.1
10.8
10.5
B1
B4
E1BCK1: RIF1
11.0
10.5
10.0
B1
B2
E1BCK1: RIF1
11.0
10.5
10.0
B1
B3
E1BCK1: RIF1
11.5
11.0
10.5
10.0
B1
B4
B: bull
replicate
R1
R2
R3
R4

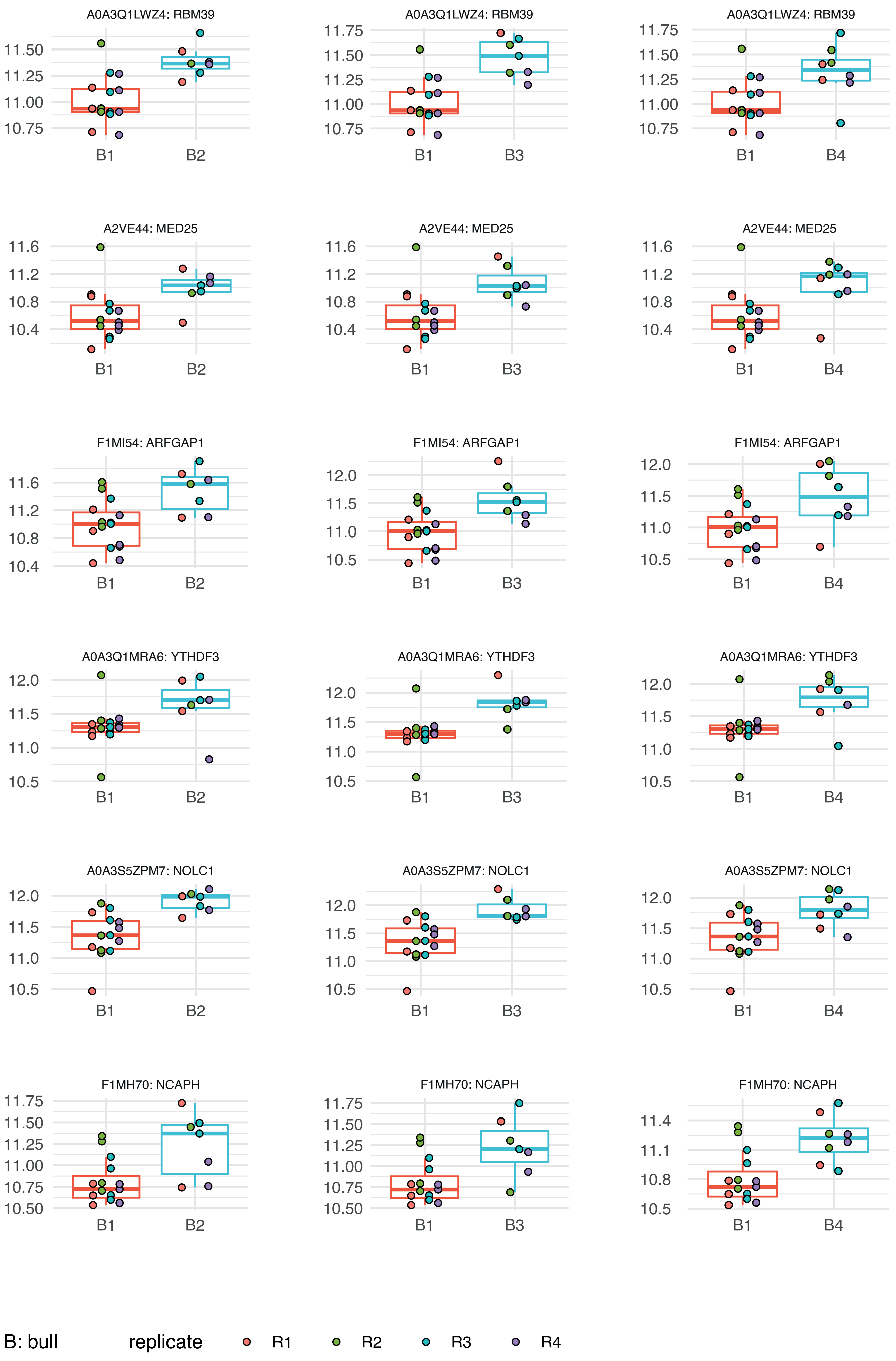
A0A3Q1LWZ4: RBM39
B1
B2
B3
B4
A2VE44: MED25
F1MI54: ARFGAP1
A0A3Q1MRA6: YTHDF3
A0A3S5ZPM7: NOLC1
F1MH70: NCAPH
B: bull
replicate
R1
R2
R3
R4

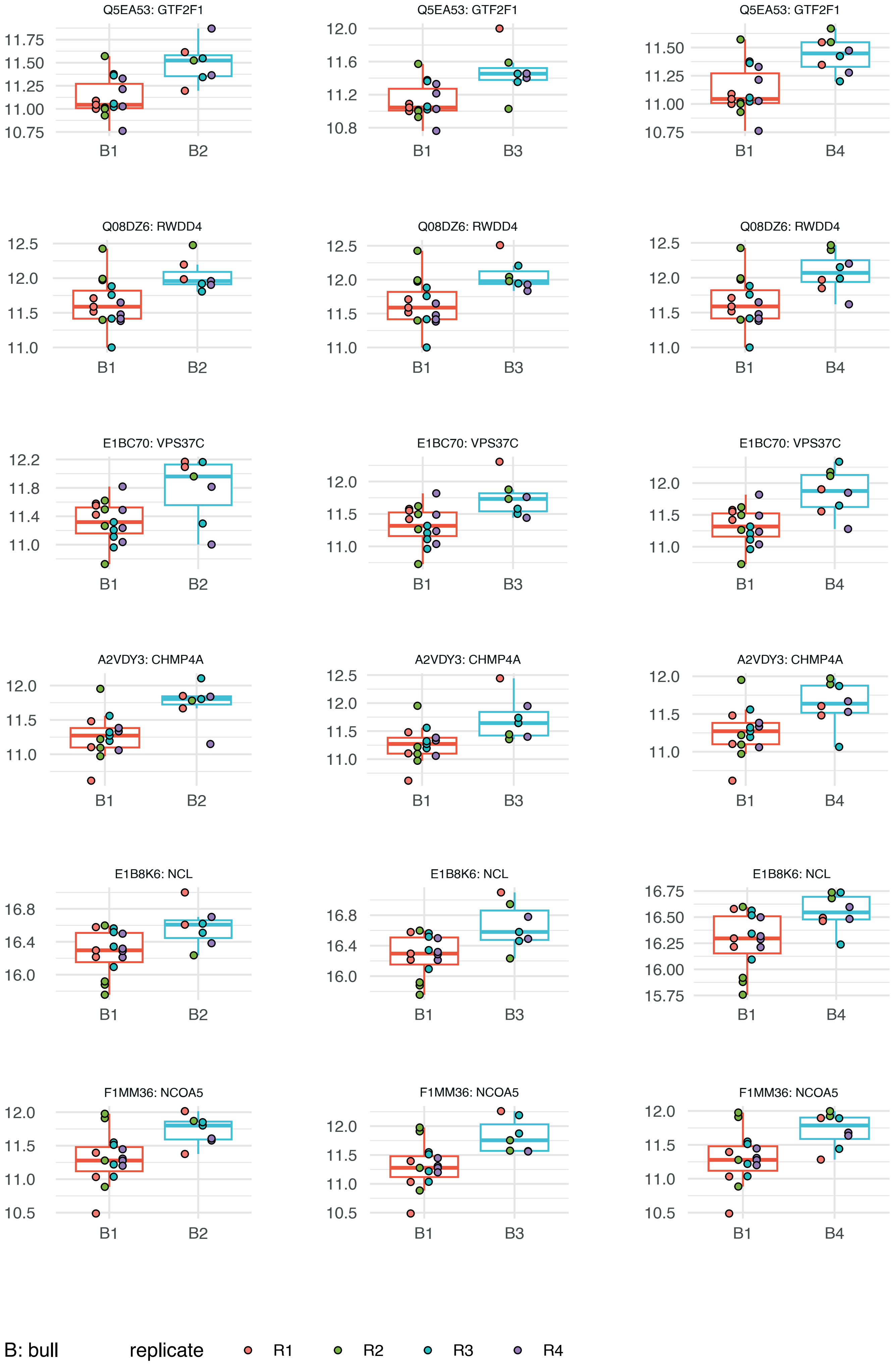
Q5EA53: GTF2F1
B1 B2
Q5EA53: GTF2F1
B1 B3
Q5EA53: GTF2F1
B1 B4
Q08DZ6: RWDD4
B1 B2
Q08DZ6: RWDD4
B1 B3
Q08DZ6: RWDD4
B1 B4
E1BC70: VPS37C
B1 B2
E1BC70: VPS37C
B1 B3
E1BC70: VPS37C
B1 B4
A2VDY3: CHMP4A
B1 B2
A2VDY3: CHMP4A
B1 B3
A2VDY3: CHMP4A
B1 B4
E1B8K6: NCL
B1 B2
E1B8K6: NCL
B1 B3
E1B8K6: NCL
B1 B4
F1MM36: NCOA5
B1 B2
F1MM36: NCOA5
B1 B3
F1MM36: NCOA5
B1 B4
B: bull
replicate R1 R2 R3 R4

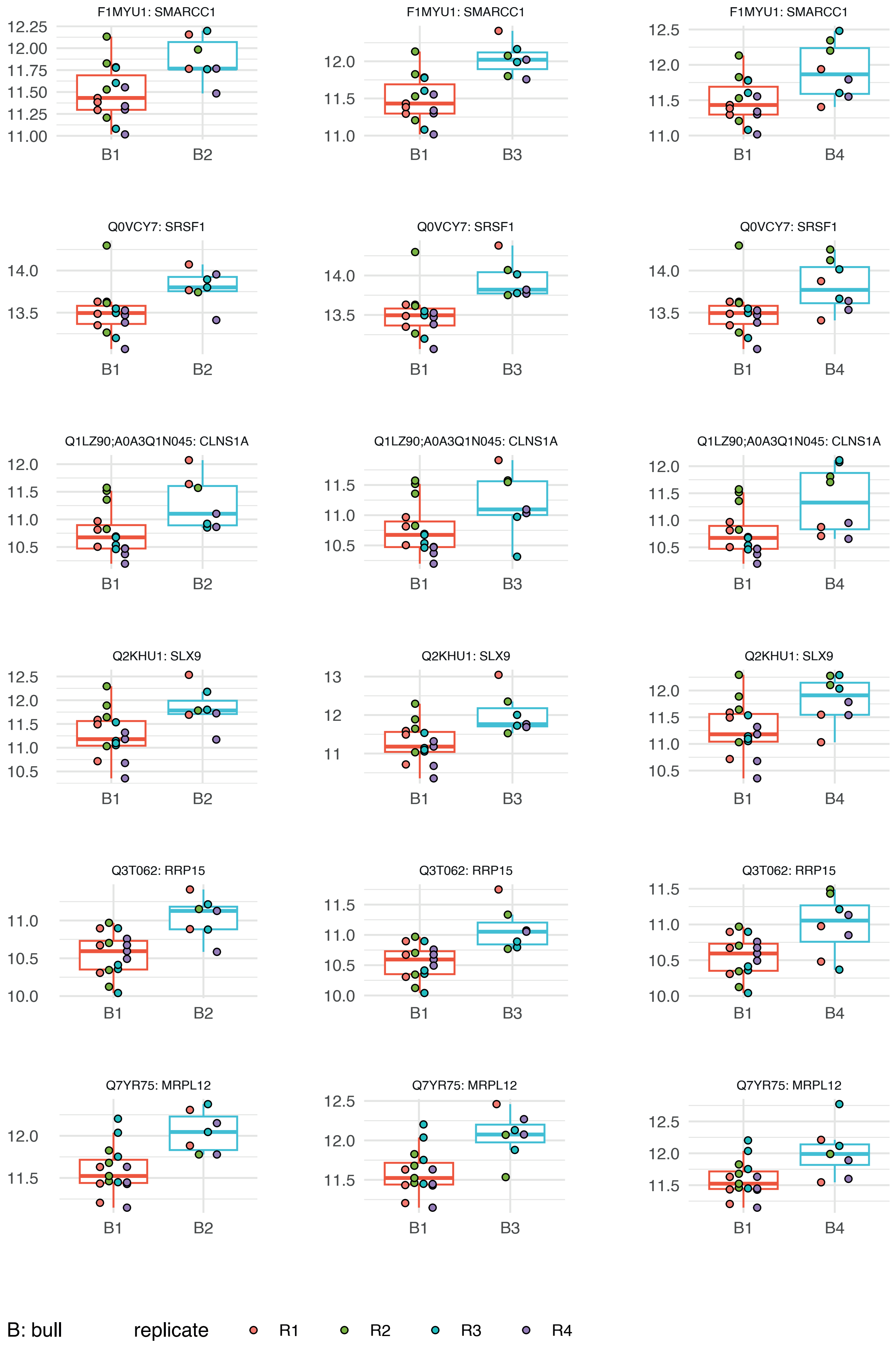
F1MYU1: SMARCC1
12.25
12.00
11.75
11.50
11.25
11.00
B1
B2
F1MYU1: SMARCC1
12.0
11.5
11.0
B1
B3
F1MYU1: SMARCC1
12.5
12.0
11.5
11.0
B1
B4
Q0VCY7: SRSF1
14.0
13.5
B1
B2
Q0VCY7: SRSF1
14.0
13.5
B1
B3
Q0VCY7: SRSF1
14.0
13.5
B1
B4
Q1LZ90;A0A3Q1N045: CLNS1A
12.0
11.5
11.0
10.5
B1
B2
Q1LZ90;A0A3Q1N045: CLNS1A
11.5
11.0
10.5
B1
B3
Q1LZ90;A0A3Q1N045: CLNS1A
12.0
11.5
11.0
10.5
B1
B4
Q2KHU1: SLX9
12.5
12.0
11.5
11.0
10.5
B1
B2
Q2KHU1: SLX9
13
12
11
B1
B3
Q2KHU1: SLX9
12.0
11.5
11.0
10.5
B1
B4
Q3T062: RRP15
11.0
10.5
10.0
B1
B2
Q3T062: RRP15
11.5
11.0
10.5
10.0
B1
B3
Q3T062: RRP15
11.5
11.0
10.5
10.0
B1
B4
Q7YR75: MRPL12
12.0
11.5
B1
B2
Q7YR75: MRPL12
12.5
12.0
11.5
B1
B3
Q7YR75: MRPL12
12.5
12.0
11.5
B1
B4
B: bull
replicate
R1
R2
R3
R4
