# Supplementary material for: Decoding bull fertility in vitro: a proteomics exploration from sperm to blastocyst
Source: Reproduction. 2025 Mar 19;169(4):e240296. doi: 10.1530/REP-24-0296 (PMC11949518; doi:10.1530/REP-24-0296)
Supplement: Supplementary file 10 [file supplementary_materials.pdf]

Sperm proteomics sample preparation

In Experiment 2 (proteomics sample collection), after addition to the IVF medium for co-incubation with the oocytes, the remaining sperm cells were washed three times in DPBS by centrifugation for 1 min at 10,000 g, and the resulting pellet (20 µL, ~ 3 x 10^8 sperm cells) was stored at -80 °C until further analysis. Individual samples (bull 1: *n* = 5, bull 2: *n* = 4, bull 3: *n* = 5, and bull 4: *n* = 3), thawed at room temperature, were lysed in 80 µL lysis buffer (10% sodium dodecyl sulfate (SAFC 8.17034, Hohenbrunn, Germany), 150 nM NaCl (Merck 106404, Darmstadt, Germany), and 100 mmol/L triethylammonium bicarbonate (TEAB; Merck 18597, Darmstadt, Germany)) for 15 min at 70 °C in a thermal mixer at 600 rpm. Samples were then transferred to a 96-well plate (Corning 3799, Kennebunk, USA) and sonicated using a PIXUL® Multi-Sample Sonicator (Active Motif, Kirkland USA) for 20 min (pulse setting = 50 N, PRF = 1.00 kHz, burst rate = 20.00 Hz). The plate was subsequently spun down for 5 min at 2,200 g and the supernatants were recovered into 1.5 mL Protein LoBind® tubes (Eppendorf 22431081, Darmstadt, Germany). These tubes were centrifuged for 15 min at 20,000 g and the supernatants were transferred to fresh 1.5 mL Protein LoBind® tubes. Proteins were reduced with 5 µL of 120 mmol/L tris(2-carboxyethyl)phosphine (TCEP-HCl, pH 7.8) (Thermo Scientific 20491, Haarlem, Netherlands) for 15 min at 55 °C and alkylated in the dark with 5 µL of 1 M 2-chloroacetamide (Sigma-Aldrich C0267, Darmstadt, Germany) for 30 min at room temperature on a thermal mixer (both at 600 rpm). The samples were subsequently processed using S-Trap™ mini columns (ProtiFi C02-mini, USA) following the manufacturer's instructions. Briefly, the samples were acidified with 10 µL 12% phosphoric acid (Sigma-Aldrich 345245, Darmstadt, Germany), loaded onto the spin columns and digested overnight with 1 µg trypsin (Promega V5111, Madison, USA) in 125 µL 50 mM TEAB at 37 °C. Peptides were eluted into 2 mL protein LoBind tubes (Eppendorf 22431102, Hamburg, Germany), dried down by SpeedVac and resuspended in 120 µL of 0.1% trifluoracetic acid (TFA) (Biosolve 202341, Dieuze, France). Methionines were oxidized with 30 µL of 3% H_2O_2 (Sigma-Aldrich 216763, Steinheim, Germany) for 30 min at 30 °C, following which the samples were cleaned up by ZipTip (Agilent A57003100BK, Ghent, Belgium) according to the manufacturer's instructions. Finally, the samples were dried in a vacuum concentrator and resuspended in 20 µL sample loading buffer (0.1% TFA, 2% acetonitrile (ACN; Fisher Chemical 10001334, Loughborough, UK)) and peptide concentrations were measured on a DropSense16 spectrophotometer (Trinean, Gentbrugge, Belgium).

Embryo proteomics sample preparation

In four replicates, embryos at the 2-4-cell stage were collected in pools of 10 embryos (bull 1: *n* = 8 pools, bull 2: *n* = 4 pools, bull 3: *n* = 4 pools, and bull 4: *n* = 4 pools), while expanded day 8 blastocysts were collected individually (bull 1: *n* = 16 embryos, bull 2: *n* = 8 embryos, bull 3: *n* = 7 embryos, and bull 4: *n* = 8 embryos). Embryos were transferred into 2 µL of DPBS in a twin.tec® PCR Plate 384 LoBind® (Eppendorf 0030129547, Hamburg, Germany) and stored frozen at -80 °C until further processing. Cells were lysed by three thaw-freeze cycles at 80°C followed by -80 °C for 15 min each, with a centrifugation at 1500 g for 10 seconds after each thawing step. Proteins were then digested overnight at 37 °C with 3 µL of digestion buffer (83.33 mmol/L TEAB, 0.33 % n-Dodecyl-B-D-maltoside (Sigma-Aldrich D4641, Steinheim, Germany), 0.0166% ProteaseMAX (Promega V2071, Madison, USA), 6.67 ng/µL Trypsin/Lys-C (Promega V5073, Madison, USA)) and then acidified with 1 µL of 5% TFA and transferred to Evotips

(Evosep EV2011, Odense, Denmark) as per manufacturer's instructions. For the sample loading step, approximately 3 µL of 0.1% TFA was aspirated into a 10 µL protein low-binding pipet tip (Socorex 3090.0010PRS, Langenhagen, Germany) prior to aspirating the entire sample, the content of the tip was then ejected into the EvoTip.

Liquid chromatography-tandem mass spectrometry analysis

For each sperm sample, 500 ng of peptide material was injected for liquid chromatography-tandem mass spectrometry (LC-MS/MS) analysis on a Vanquish™ Neo UHPLC System in-line connected to an Orbitrap Exploris 240 mass spectrometer (Thermo Scientific, Bremen, Germany). Injection was performed in a trap-and-elute workflow using a 5 mm trapping column (Thermo scientific, 300 µm internal diameter (I.D.), 5 µm C18 beads) and a 250 mm Aurora Ultimate, 1.7 µm C18 beads, 75 µm I.D. analytical column (Ionopticks, Fitzroy, Australia) kept at a constant temperature of 45 °C. Peptides were eluted by a gradient starting at 0.5% MS strong wash solvent (SW) (0.1% formic acid (Merck 100264, Darmstadt, Germany) (FA) in ACN) reaching 26% MS SW in 75 min, 44% MS SW in 95 min, 56% MS SW in 100 min followed by 5 min wash at 56% MS SW and column equilibration. The mass spectrometer was operated in data-independent mode, automatically switching between MS and MS/MS acquisition. Full-scan MS spectra ranging from m/z 400-900, with a precursor isolation width of 10 Th, MS2 spectra were acquired within a scan range of m/z 200-1800. EASY-IC™ was used at the start of the run as Internal Mass Calibration and QCloud has been used to control instrument longitudinal performance during the project (Chiva et al., 2018; Olivella et al., 2021).

Embryo samples were run in data-independent parallel accumulation serial fragmentation (DIA-PASEF) mode on an Evosep One LC-system (Evosep, Denmark) in-line connected to a timsTOF SCP mass spectrometer (Bruker, Bremen, Germany). Peptides were analyzed with the 20 SPD whisper method using the Aurora Gen3 Elite column (15cm x 75 µm I.D., 1.7 µm C18 beads, Ionopticks, Fitzroy, Australia), heated to 50 °C. Peptides were eluted from the column through the predefined 20 SPD whisper gradient consisting of 0.1% FA in LC-MS-grade water as solvent A, and 0.1% FA in ACN as solvent B. Eluting peptides were measured in positive polarity with a full-scan range of m/z 100 to 1700. The TIMS was operated at a fixed duty cycle close to 100%, a ramp and accumulation time of 100 ms, ranging from $1/K_0$ =0.64 Vscm^2 to $1/K_0$ =1.50 Vscm^2. Collision energy was linearly ramped as a function of the inverse mobility from 20 eV at $1/K_0$= 0.60 Vscm^2 to 59 eV at $1/K_0$ = 1.60 Vscm^2. A DIA-PASEF mass range of 400 to 1000 Th was used in a mobility range of $1/K_0$ =0.64 Vscm^2 to $1/K_0$ =1.37 Vscm^2 using a window size of 25 Th according to Table S1, resulting in a cycle time of 0.96 s.

Proteomics data analysis

Sperm raw data files were searched using DIA-NN v1.8.1 (Demichev et al., 2020, 2022). The search engine was supplied with the bovine reference proteome (*Bos taurus* UP000009136 with 23,836 entries; one protein sequence per gene) for library generation, allowing for one missed cleavage using Trypsin/P as the protease, up to two variable modifications, with N-terminal methionine excision, methionine oxidation, and N-terminal acetylation set as variable modifications and cysteine carbamidomethylation set as a fixed modification. Peptide length and charge ranges were set to 7-30 and 1-4, respectively. Precursor and fragment ion ranges were set to respectively 400-900 and 200-1800. Precursors were

filtered at 1% FDR, with robust LC (high accuracy), cross-run retention time-based normalization and match between runs enabled.

Embryo raw data files were searched using identical settings as described above, with the following exceptions: cysteine carbamidomethylation was disabled, the precursor mass range was set to 400-1000, and robust LC (high precision) was enabled.

Downstream data analysis was performed with R version 4.3.3 within the RStudio environment version 2023.12.0+369. Statistical analysis was performed using the MSqRob2 and QFeatures packages (Gatto L & Vanderaa C, 2024; Goeminne et al., 2020). Plotting was performed using the ggplot2 package (Wickham, 2016). Gene set enrichment analysis (GSEA) analyses were performed using the WebGestaltR package (Liao et al., 2019). Briefly, non-proteotypic peptides were filtered out and only proteins for which at least two different peptides were identified across each respective dataset were considered for downstream analysis. Furthermore, only peptides which were identified in at least two (sperm and 2-cell stage) or four (blastocysts) technical replicates per bull were retained for the differential expression analysis. Peptide intensities were log_2-transformed, median-normalized and then aggregated into protein abundances using the median polish procedure implemented in MSqRob2. Differential expression analysis was performed using MSqRob2. P-values were adjusted using the Benjamini-Hochberg method with an FDR of 0.05.

References

Chiva, C., Olivella, R., Borràs, E., Espadas, G., Pastor, O., Solé, A. & Sabidó, E. (2018). QCloud: A cloud-based quality control system for mass spectrometry-based proteomics laboratories. PloS One, 13(1). https://doi.org/10.1371/JOURNAL.PONE.0189209

Demichev, V., Messner, C. B., Vernardis, S. I., Lilley, K. S. & Ralser, M. (2020). DIA-NN: neural networks and interference correction enable deep proteome coverage in high throughput. Nature Methods, 17(1), 41–44. https://doi.org/10.1038/S41592-019-0638-X

Demichev, V., Szyrwiel, L., Yu, F., Teo, G. C., Rosenberger, G., Niewienda, A., Ludwig, D., Decker, J., Kaspar-Schoenefeld, S., Lilley, K. S., Mülleder, M., Nesvizhskii, A. I. & Ralser, M. (2022). dia-PASEF data analysis using FragPipe and DIA-NN for deep proteomics of low sample amounts. Nature Communications, 13(1). https://doi.org/10.1038/S41467-022-31492-0

Gatto L & Vanderaa C. (2024). Quantitative features for mass spectrometry data. R package version 1.14.1. Bioconductor. https://bioconductor.org/packages/release/bioc/html/QFeatures.html

Goeminne, L. J. E., Sticker, A., Martens, L., Gevaert, K. & Clement, L. (2020). MSqRob takes the missing hurdle: uniting intensity- and count-based proteomics. Analytical Chemistry, 92(9), 6278–6287. https://doi.org/10.1021/ACS.ANALCHEM.9B04375/SUPPL_FILE/AC9B04375_SI_006.XLSX

Liao, Y., Wang, J., Jaehnig, E. J., Shi, Z. & Zhang, B. (2019). WebGestalt 2019: gene set analysis toolkit with revamped UIs and APIs. Nucleic Acids Research, 47(W1), W199–W205. https://doi.org/10.1093/NAR/GKZ401

Olivella, R., Chiva, C., Serret, M., Mancera, D., Cozzuto, L., Hermoso, A., Borràs, E., Espadas, G., Morales, J., Pastor, O., Solé, A., Ponomarenko, J. & Sabidó, E. (2021). QCloud2: an improved cloud-based quality-control system for mass-spectrometry-based proteomics laboratories. Journal of Proteome Research, 20(4), 2010–2013. https://doi.org/10.1021/ACS.JPROTEOME.0C00853

Wickham, H. (2016). ggplot2: Elegant graphics for data analysis. https://doi.org/10.1007/978-3-319-24277-4
